# FibroScan-AST (FAST) score for the non-invasive identification of patients with non-alcoholic steatohepatitis with significant activity and fibrosis: a prospective derivation and global validation study

**DOI:** 10.1016/S2468-1253(19)30383-8

**Published:** 2020-02-03

**Authors:** Philip N Newsome, Magali Sasso, Jonathan J Deeks, Angelo Paredes, Jérôme Boursier, Wah-Kheong Chan, Yusuf Yilmaz, Sébastien Czernichow, Ming-Hua Zheng, Vincent Wai-Sun Wong, Michael Allison, Emmanuel Tsochatzis, Quentin M Anstee, David A Sheridan, Peter J Eddowes, Indra N Guha, Jeremy F Cobbold, Valérie Paradis, Pierre Bedossa, Véronique Miette, Céline Fournier-Poizat, Laurent Sandrin, Stephen A Harrison

**Affiliations:** a National Institute for Health Research Biomedical Research Centre at University Hospitals Birmingham NHS Foundation Trust and the University of Birmingham, Birmingham, UK; b Centre for Liver and Gastrointestinal Research, Institute of Immunology and Immunotherapy, University of Birmingham, Birmingham, UK; c Test Evaluation Research Group, Institute of Applied Health Research, University of Birmingham, Birmingham, UK; d Liver Unit, University Hospitals Birmingham NHS Foundation Trust, Birmingham, UK; e R&D Department, Echosens, Paris, France; f Medical Affairs Department, Echosens, Paris, France; g Brooke Army Medical Center, Fort Sam Houston, TX, USA; h Uniformed Services University of the Health Sciences, Bethesda, MD, USA; i Department of Hepato-Gastroenterology, Angers University Hospital, Angers, France; j HIFIH Laboratory UPRES EA3859, SFR4208, Angers University, Angers, France; k Gastroenterology and Hepatology Unit, Gastrointestinal Endoscopy Unit, Department of Medicine, Faculty of Medicine, University of Malaya, Kuala Lumpur, Malaysia; l Liver Research Unit, Institute of Gastroenterology, Marmara University, Istanbul, Turkey; m Department of Gastroenterology, School of Medicine, Marmara University, Istanbul, Turkey; n Department of Nutrition, Hôpital Européen Georges-Pompidou (APHP), University of Paris, Paris, France; o NAFLD Research Center, Department of Hepatology, the First Affiliated Hospital of Wenzhou Medical University, Wenzhou, China; p Institute of Hepatology, Wenzhou Medical University, Wenzhou, China; q Department of Medicine and Therapeutics, The Chinese University of Hong Kong, Hong Kong, China; r Department of Medicine, Cambridge Biomedical Research Centre, Cambridge University Hospitals NHS Foundation Trust, Cambridge, UK; s UCL Institute for Liver and Digestive Health, Royal Free Hospital and UCL, London, UK; t Institute of Cellular Medicine–Faculty of Medical Sciences, Newcastle University, Newcastle upon Tyne, UK; u Newcastle NIHR Biomedical Research Centre, Newcastle upon Tyne Hospitals NHS Trust, Newcastle upon Tyne, UK; v Institute of Translational and Stratified Medicine, University of Plymouth, Plymouth, UK; w NIHR Nottingham Biomedical Research Centre, Nottingham University Hospitals NHS Trust and the University of Nottingham, Nottingham, UK; x NIHR Oxford Biomedical Research Centre and Oxford Liver Unit, Oxford University Hospitals NHS Foundation Trust, John Radcliffe Hospital, Oxford, UK; y Pathology Department, Hôpital Beaujon, APHP, Clichy, France; z Liverpat, Paris, France; aa Radcliffe Department of Medicine, University of Oxford, Oxford, UK

## Abstract

**Background:**

The burden of non-alcoholic fatty liver disease (NAFLD) is increasing globally, and a major priority is to identify patients with non-alcoholic steatohepatitis (NASH) who are at greater risk of progression to cirrhosis, and who will be candidates for clinical trials and emerging new pharmacotherapies. We aimed to develop a score to identify patients with NASH, elevated NAFLD activity score (NAS≥4), and advanced fibrosis (stage 2 or higher [F≥2]).

**Methods:**

This prospective study included a derivation cohort before validation in multiple international cohorts. The derivation cohort was a cross-sectional, multicentre study of patients aged 18 years or older, scheduled to have a liver biopsy for suspicion of NAFLD at seven tertiary care liver centres in England. This was a prespecified secondary outcome of a study for which the primary endpoints have already been reported. Liver stiffness measurement (LSM) by vibration-controlled transient elastography and controlled attenuation parameter (CAP) measured by FibroScan device were combined with aspartate aminotransferase (AST), alanine aminotransferase (ALT), or AST:ALT ratio. To identify those patients with NASH, an elevated NAS, and significant fibrosis, the best fitting multivariable logistic regression model was identified and internally validated using boot-strapping. Score calibration and discrimination performance were determined in both the derivation dataset in England, and seven independent international (France, USA, China, Malaysia, Turkey) histologically confirmed cohorts of patients with NAFLD (external validation cohorts). This study is registered with ClinicalTrials.gov, number NCT01985009.

**Findings:**

Between March 20, 2014, and Jan 17, 2017, 350 patients with suspected NAFLD attending liver clinics in England were prospectively enrolled in the derivation cohort. The most predictive model combined LSM, CAP, and AST, and was designated FAST (FibroScan-AST). Performance was satisfactory in the derivation dataset (C-statistic 0·80, 95% CI 0·76–0·85) and was well calibrated. In external validation cohorts, calibration of the score was satisfactory and discrimination was good across the full range of validation cohorts (C-statistic range 0·74–0·95, 0·85; 95% CI 0·83–0·87 in the pooled external validation patients' cohort; n=1026). Cutoff was 0·35 for sensitivity of 0·90 or greater and 0·67 for specificity of 0·90 or greater in the derivation cohort, leading to a positive predictive value (PPV) of 0·83 (84/101) and a negative predictive value (NPV) of 0·85 (93/110). In the external validation cohorts, PPV ranged from 0·33 to 0·81 and NPV from 0·73 to 1·0.

**Interpretation:**

The FAST score provides an efficient way to non-invasively identify patients at risk of progressive NASH for clinical trials or treatments when they become available, and thereby reduce unnecessary liver biopsy in patients unlikely to have significant disease.

**Funding:**

Echosens and UK National Institute for Health Research.

## Introduction

Non-alcoholic fatty liver disease (NAFLD) is rising in prevalence along with levels of obesity and type 2 diabetes, such that it is now the most common cause of chronic liver disease worldwide.^1^ Prevalence in the general population is 25–35%, but this rises to 70% in patients with obesity and type 2 diabetes.^1^ Although most patients with NAFLD do not progress to advanced fibrosis or cirrhosis, the high prevalence of NAFLD means that many patients do develop chronic liver disease, and that NAFLD is now one of main indications for liver transplantation in Europe^2^ and the USA.^3^ A key challenge is the identification of patients who are at greatest risk of clinical progression by way of worsening liver fibrosis, and who might benefit from treatment with new pharmacotherapies.^4^, ^5^

**Research in context**

**Evidence before this study**

Existing diagnostic scores focus only on fibrosis and have not been successful in enhancing screen failure rates in clinical trials. The concept of identifying patients with non-alcoholic steatohepatitis, an elevated non-alcoholic fatty liver disease activity score, and F2 fibrosis for the purpose of inclusion in clinical trials had not been previously considered. PubMed searches for those terms up to September, 2019, with no language restrictions, did not reveal any publications in this area. Previous studies had only focused on identifying patients with fibrosis but not concomitant evaluation of inflammation.

**Added value of this study**

This study provides a solution to better identify patients who might be candidates for clinical trials or treatments as they become available. There is a high screen failure rate at liver biopsy because of the absence of such tools. This score will reduce the number of patients having unnecessary liver biopsy.

**Implications of all the available evidence**

The results of this study have the potential to help clinicians and investigators in their decision making, to better select patients for clinical trials or access to emerging therapies, and streamline the need for liver biopsies among patients with non-alcoholic steatohepatitis in clinical care, probably resulting in a reduction in the number of biopsies required. Future research should focus on studying the performance of the FAST (FibroScan-AST) score in primary care.

Currently, identification of patients with active NASH and significant fibrosis can be done using non-invasive markers that risk-stratify liver fibrosis^6^ or require percutaneous liver biopsy. The use of non-invasive markers includes algorithms,^7^ serum biomarkers,^8^ and imaging modalities,^9^ but makes no determination of the presence or degree of inflammatory liver injury. The presence of non-alcoholic steatohepatitis (NASH) and more profound liver cell injury, as determined by measures of steatosis (lobular inflammation and ballooning),^10^ are crucial drivers of the development of liver fibrosis in patients with NAFLD and will be important in risk stratification.

We present a prespecified secondary analysis of a previously published study,^11^ with the aim of developing an algorithm to diagnose, among people with suspected NAFLD, those with NASH, significant liver fibrosis (≥F2), and elevated NAFLD activity score (NAS≥4). This combination of criteria is important, as the presence of fibrosis alone is insufficient for recruitment to clinical trials. Moreover, the presence of inflammation defined by NASH and elevated NAS will be important in identifying patients who could benefit from anti-inflammatory therapies, as these interventions might not be as relevant for patients with fibrosis but no or minimal inflammatory injury. Studies have shown that histological responses to trial medications are more common in patients with elevated NAS.^12^ We aimed to develop an algorithm to identify this subgroup of patients for clinical trial eligibility and for optimal prescription of therapies.

## Methods

### Study design

**T**his prospective study was done as a derivation cohort before validation in multiple global cohorts (named external validation cohorts hereafter). The transparent reporting of a multivariable prediction model for individual prognosis or diagnosis (TRIPOD) guidelines^13^ were followed to report the development and internal and external validation of the prediction model for diagnosis of NASH + NAS ≥ 4 + F ≥ 2 (appendix p 3).^14^

The derivation cohort was a cross-sectional, prospective, multicentre study. Consecutive patients were recruited from seven tertiary care liver centres across England (University Hospitals Birmingham NHS Foundation Trust, Birmingham; Addenbrooke's Hospital, Cambridge; Royal Free Hospital, London; Freeman Hospital, Newcastle upon Tyne; University Hospitals Plymouth NHS Trust, Plymouth; Queen's Medical Centre, Nottingham; and John Radcliffe Hospital, Oxford). The study was approved by the North Wales Research Ethics Committee (13/WA/0385) and by the local research ethics committee at each centre. The study was done in accordance with the Declaration of Helsinki and in agreement with the International Conference on Harmonisation guidelines on Good Clinical Practice. The primary objective of this study was to assess the diagnostic accuracy of controlled attenuation parameter (CAP) and secondary objectives were to assess the diagnostic accuracy of liver stiffness measurement (LSM) by vibration-controlled transient elastography (VCTE), comparing CAP and LSM by VCTE with other non-invasive tests and also to develop a score combining LSM by VCTE, CAP, and biological markers to diagnose NASH. Results for the identification of steatosis and fibrosis were reported by Eddowes and colleagues,^11^ whereas we report the prespecified secondary objective of the development of a score to identify patients with NASH and significant liver cell injury and fibrosis.

### Participants

Eligible patients were aged 18 years or older, able to give written informed consent, and scheduled (independently from this study) to have a liver biopsy for investigation of suspected NAFLD (usually as a result of abnormal liver enzymes and an ultrasound scan showing an echobright liver) within 2 weeks before or after LSM by VCTE and CAP measurements. All patients gave written informed consent to participate in the study. Eligible patients were negative for hepatitis B surface antigen, anti-hepatitis C virus antibody, hepatitis C virus RNA, and hepatitis B virus DNA. Patients were excluded in case of ascites, pregnancy, active implantable medical device (such as pacemaker or defibrillator), liver transplantation, cardiac failure or clinically significant valvular disease, haemochromatosis, refusal to have liver biopsy or blood tests, alcohol consumption above recommended limits (>14 units per week for women and >21 units per week for men), diagnosis of active malignancy or other terminal disease, or participation in another clinical trial within the previous 30 days. Age, sex, body-mass index (BMI), and presence of diabetes, hypertension, and hypercholesterolaemia were recorded for each patient. A 12 h fasting blood sample was obtained locally and then shipped to a central laboratory for assessment.

### Procedures

Percutaneous liver biopsy was done in all patients and used as the reference. Specimens were fixed in formalin, embedded in paraffin, and stained with haematoxylin and eosin and Picrosirius red. Slides were analysed independently by two experienced pathologists (PB, VP) masked to each other's reading, and to the patient's clinical and FibroScan data. In case of disagreement, they reviewed the slides together to reach consensus. Steatosis, ballooning, lobular inflammation grades, fibrosis stage, and NAS were assessed using the NASH Clinical Research Network (CRN) scoring system.^10^ NASH was diagnosed using the fatty liver: inhibition of progression (FLIP) definition (at least grade one for steatosis, ballooning, and lobular inflammation).^15^

LSM by VCTE and CAP were both measured using FibroScan 502 Touch devices equipped with both M and XL probes (Echosens, Paris, France) by nurses or physicians trained and certified by the manufacturer and masked to the patient's histological evaluation.^16^, ^17^ Patients were asked to fast for at least 3 h before the examination. Probe selection was done using the automatic probe selection tool embedded in the device software. Patients were placed in the supine position with their right arm fully abducted, and measurements were done by scanning the right liver lobe through an intercostal space. CAP is an average estimate of ultrasound attenuation at 3·5 MHz and is expressed in dB/m. LSM by VCTE is an average estimate of stiffness (Young's modulus) at a shear wave frequency of 50 Hz and is expressed as kPa. Only examinations with at least ten valid individual measurements were deemed valid.

### Outcomes and predictor variables

The main outcome was the diagnosis of NASH (using FLIP definition) with NAS 4 or higher, and fibrosis stage 2 or higher (NAS and fibrosis stage scored using CRN scoring system; combination of criteria hereafter referred to as NASH + NAS ≥ 4 + F ≥ 2). The models considered five predictor variables: LSM by VCTE, CAP, aspartate aminotransferase (AST), alanine aminotransferase (ALT) or AST:ALT ratio (AAR). We anticipated that only one of AST, ALT, and AAR would be included in the final model.

### External validation cohorts

Data from seven clinical studies were gathered to perform the external validation of the score. Data came from North America, Europe, and Asia. Five cohorts came from tertiary care liver centres, one cohort came from a bariatric surgery centre, and one cohort came from a study of screening for NAFLD in patients having a routine colonoscopy. All external validation cohort data were collected in the framework of a clinical study for which the local ethical committee granted approval. All patients from each study gave written informed consent to participate in the study. Each study was done in accordance with the Declaration of Helsinki and in agreement with the International Conference on Harmonisation guidelines on Good Clinical Practice. Enrolment dates of each study are shown in the appendix (p 5) together with descriptions of external validation cohorts. Datasets were locked at the date of the last inclusion (appendix p 5). However, the Chinese Wenzhou, French, and Turkish NAFLD studies were ongoing at database lock (Wenzhou was expected to end in December, 2022, the French open registry had no planned termination date, and the Turkish study terminated in May, 2019). In each external validation study, patients were recruited consecutively, FibroScan operators were masked to patients' clinical data, and all liver biopsy results were read by expert pathologists who were masked to patient clinical data and FibroScan device results. For the two studies that had all patients measured with both M and XL probes (Chinese Hong-Kong and French NAFLD cohorts), the FibroScan examination corresponding to the XL probe was considered if the patient's BMI was greater or equal to 32 kg/m^2^; otherwise the M probe was considered.

All external validation cohorts excluded patients who met the following criteria: non-metabolic comorbidities that could have induced liver lesions such as viral hepatitis, drug-induced liver injury, excessive alcohol consumption, or HIV; BMI higher than 32 kg/m^2^ if only the M probe was available for the study; less than ten valid measurements for FibroScan; missing data for the developed score; liver biopsy results that were non-interpretable or with missing data for the target of NASH + NAS ≥ 4 + F ≥ 2; or a time interval greater than 1 year between FibroScan and liver biopsy. In each external validation cohort, patients had their individual lesions of steatosis, lobular inflammation, ballooning grades, and fibrosis stage scored according to the NASH CRN scoring system.^10^ From these individual items, a diagnosis of NASH was made according to the FLIP definition,^15^ and the NASH + NAS ≥ 4 + F ≥ 2 outcome was computed.

### Statistical analysis

Sample size was determined for the primary objective of estimating the accuracy of CAP to achieve a 5% standard error in the estimates of the area under the receiver operating curve (AUROC) parameter in the subgroup using the XL probe. Assuming two-thirds of recruited patients would use the XL probe, and allowing for 30% dropout, the target sample size was set at 450. This sample size was judged adequate to provide robust estimates for predictive models based on five covariates, with over 30 events per variable at an expected prevalence of 50%. The score was developed on the 350 patients in the derivation cohort. Eight (2%) of 350 patients had missing data for CAP, AST, or ALT, and since the proportion of observations with missing data was less than 3%, single imputation was done using stochastic regression imputation.^18^, ^19^ The selection of parameters was based on the combination of LSM by VCTE (related to liver fibrosis), and CAP (related to liver steatosis), with factors linked to NASH, inflammation, and fibrosis (AST, ALT, AAR). Parameters were combined into a multivariable logistic regression model. Akaike's information criterion was used to select between AST, ALT, and AAR as the optimal parameter to combine to LSM by VCTE and CAP. The relative importance of each parameter was appraised using the Wald test. Nested models were compared using the likelihood ratio test. Optimal exploratory variable transformations were selected using multivariable first-degree fractional polynomials to optimise the model.^20^ In this method, first order fractional polynomials are formulated as a power transformation of the predictors taken from the set −2, −1, −0·5, 0, 0·5, 1, 2, 3. Optimal power is selected for each predictor (considered in order of decreasing statistical significance) using a backward stepwise selection procedure.^20^

The model was internally validated using 2000 bootstrap samples.^19^ Within each bootstrap iteration, we refitted the model and evaluated the performance in the bootstrap sample (apparent performance) and in the original data (test performance). Performance was assessed in terms of AUROC. The optimism was quantified as the mean differences of the performance estimates, and the shrinkage factor computed and applied to each regression coefficient in the original model to adjust the model for overfitting. Alternative methods such as cross-validation could have been used to perform the internal validation of the score, but bootstrapping is considered superior to cross-validation as it allows quantification of the extent of overfitting and optimism.^13^ Bootstrapping also provides an estimate of a correction factor (called shrinkage factor) that can be used to adjust the regression coefficients for overfitting such that better performance will be obtained when applying the score to new patients.

Model performance was assessed by calibration and discrimination in both the derivation and validation cohorts. Calibration (the agreement between observed outcomes and prediction) was assessed using calibration plots and a smoothing technique based on locally estimated scatterplot smoothing (Loess).^19^ Discrimination was assessed using AUROC (similar to Harrell's C-statistic). Cutoffs for sensitivity (≥0·90) and specificity (≥0·90) were derived in the derivation cohort. When appraising performance at a given cutoff, sensitivity, specificity, positive predictive value (PPV), and negative predictive value (NPV) were computed together with 95% CIs (see appendix p 7 for potential risk of bias in each external validation cohort). AUROC comparison was done using the Delong test. Statistical analyses were done using the software R, version 3.4.1.^21^ This study is registered with ClinicalTrials.gov, NCT01985009.

### Role of the funding source

The funder of the study had a role in study design, data collection, data analysis, data interpretation and writing of the report. The corresponding author and the funder had full access to all data in the study and had full responsibility for the decision to submit the publication.

## Results

**Figure 1:**
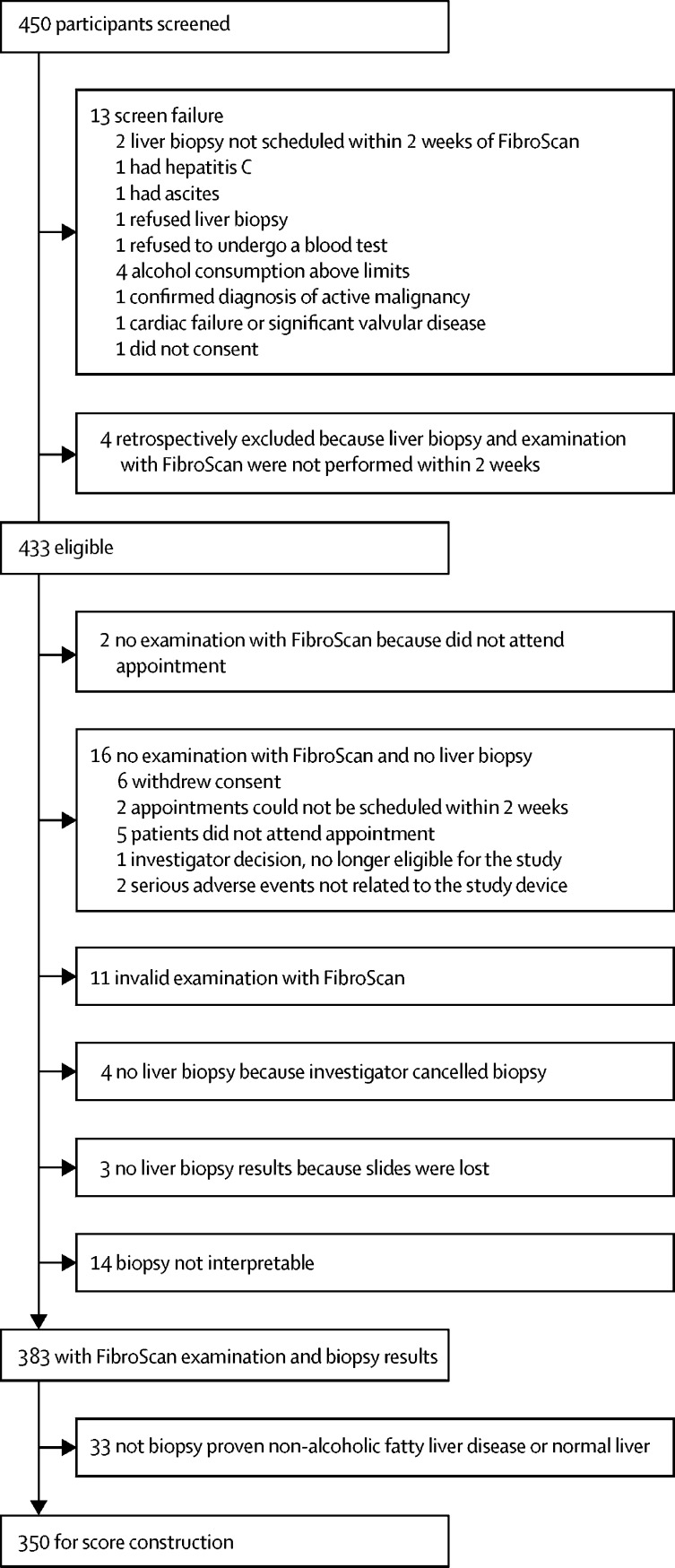
Derivation cohort trial profile

Between March 20, 2014, and Jan 17, 2017, we assessed 450 potentially eligible participants (figure 1), 350 of whom were included in the FibroScan-AST (FAST) score construction, which was subsequently validated in seven cohorts (total of 1026 patients). As reported in table 1, the derivation cohort had broadly similar demographic, metabolic, serological, and histological characteristics to patients in the pooled validation cohorts. NASH was reported in 241 (69%) of 350 patients in the derivation cohort and 595 (58%) of 1026 patients in the pooled validation cohort. NASH + NAS ≥ 4 + F ≥ 2 was reported in 174 (50%) of 350 patients in the derivation cohort and 277 (27%) of 1026 patients in the pooled validation cohort.

**Table 1:** Derivation and external validation cohort patient characteristics

		**Derivation cohort**	**French bariatric surgery cohort**	**USA screening cohort**	**China Hong-Kong NAFLD cohort**	**China Wenzhou NAFLD cohort**	**French NAFLD cohort**	**Malaysian NAFLD cohort**	**Turkish NAFLD cohort**	**Pooled external patients cohort**
**Demographics**										
n		350	110	242	83	104	182	176	129	1026
Age (years)		54 (45–63)	41 (33–50)	55 (50–60)	55 (46–63)	41 (30–50)	58 (49–66)	52 (46–60)	49 (38–57)	52 (44–60)
Female		149 (43%)	88 (80%)	97 (40%)	42 (51%)	28 (27%)	65 (36%)	84 (48%)	59 (46%)	463 (45%)
Male		201 (57%)	22 (20%)	145 (60%)	41 (49%)	76 (73%)	117 (64%)	92 (52%)	70 (54%)	563 (55%)
BMI (kg/m^2^)		34·2 (29·6–38·6)	43·0 (38·8–47·2)	32·6 (30·0–36·1)	28·9 (26·0–31·9)	25·5 (23·4–27·6)	31·6 (28·6–37·2)	28·1 (25·9–30·0)	33·0 (30·0–36·0)	31·0 (27·7–36·1)
**Metabolic**										
Diabetes (type 1 and 2)		176 (50%)	25 (23%)	55 (23%)	54 (65%)	26 (25%)	86 (47%)	90 (51%)	79 (61%)	415 (40%)
Hypertension		189 (54%)	29 (26%)	113 (47%)	57 (69%)	17 (16%)	..	104 (59%)	69 (53%)	389 (46%)
**Blood**										
AST (IU/L)		36 (27–52)	26 (21–39)	22 (18–27)	41 (28–59)	34 (27–52)	36 (28–50)	38 (29–62)	37 (28–59)	32 (23–48)
ALT (IU/L)		50 (34–72)	37 (31–54)	25 (19–38)	65 (32–97)	48 (32–88)	48 (32–77)	63 (43–104)	54 (34–106)	44 (28–74)
GGT (IU/L)		57 (34–113)	36 (23–52)	27 (20–40)	56 (37–90)	50 (28–77)	68 (37–131)	74 (41–122)	54 (34–86)	46 (28–85)
Albumin (g/L)		4·5 (4·3–4·7)	3·9 (3·7–4·1)	4·3 (4·1–4·5)	4·3 (3·9–4·6)	4·8 (4·5–5·0)	4·3 (4·0–4·5)	4·3 (4·1–4·6)	4·6 (4·3–4·8)	4·4 (4·1–4·6)
Platelets count (x 10^9^/L)		239 (199–281)	247 (216–283)	236 (201–285)	225 (178–263)	237 (206–266)	223 (170–269)	272 (228–316)	222 (190–267)	238 (199–284)
Fasting glucose (mg/dL)		108 (91–142)	88 (79–103)	103 (93–120)	117 (99–141)	92 (86–108)	111 (99–141)	105 (94–128)	109 (96–126)	104 (92–125)
Triglyceride (mg/dL)		163 (119–213)	130 (100–170)	135 (94–190)	150 (115–221)	178 (116–272)	..	133 (106–168)	169 (116–227)	146 (106–197)
Total cholesterol (mg/dL)		181 (147–212)	192 (167–221)	190 (158–217)	181 (154–207)	184 (150–217)	..	181 (162–216)	214 (182–242)	190 (161–220)
HDL cholesterol (mg/dL)		42 (34–50)	50 (40–60)	48 (39–58)	46 (39–50)	36 (33–42)	..	45 (39–52)	44 (39–53)	45 (38–54)
**Fibrosis scores**										
FIB-4		1·13 (0·78–1·68)	0·69 (0·48–1·11)	0·99 (0·81–1·31)	1·27 (0·96–1·72)	0·91 (0·62–1·20)	1·38 (0·90–1·96)	0·96 (0·65–1·40)	1·17 (0·79–1·59)	1·04 (0·72–1·46)
NFS		−1·00 (−2·12 to 0·08)	−0·80 (−2·17 to 0·08)	−0·97 (−1·90 to 0·04)	−0·95 (−2·04 to 0·16)	−2·77 (−3·61 to 1·87)	−0·60 (−1·38 to 0·57)	−2·16 (−3·04 to 1·17)	−1·12 (−1·80 to 0·22)	−1·28 (−2·32 to 0·24)
**FibroScan**										
Probe size										
	M	111 (32%)	10 (9%)	141 (58%)	63 (76%)	104 (100%)	99 (54%)	176 (100%)	68 (53%)	661 (64%)
	XL	239 (68%)	100 (91%)	101 (42%)	20 (24%)	0	83 (46%)	0	61 (47%)	365 (36%)
LSM by VCTE (kPa)		8·9 (6·2–13·9)	5·9 (4·7–8·8)	6·0 (4·7–8·2)	8·8 (6·6–12·2)	5·8 (5·1–6·7)	7·9 (5·9–11·5)	7 (6–10)	11·1 (8·6–14·6)	7·2 (5·3–10·3)
CAP (dB/m)		342 (307–373)	310 (275–374)	317 (276–360)	319 (290–354)	316 (284–332)	326 (297–369)	323 (289–343)	329 (304–356)	321 (288–355)
**Histology**										
Length of liver biopsy specimen (mm)		23 (10)	12 (5)	14 (5)	23 (8)	..	29 (11)	15 (4)	30 (14)	17 (12)
Length of liver biopsy specimen ≥15 mm		315 (90%)	50 (45%)	109 (45%)	74 (89%)	..	169 (93%)	102 (58%)	125 (98%)	629 (68%)
**Fibrosis stage**										
0		60 (17%)	65 (59%)	131 (54%)	9 (11%)	45 (43%)	28 (15%)	62 (35%)	16 (12%)	356 (35%)
1		80 (23%)	26 (24%)	74 (31%)	23 (28%)	46 (44%)	46 (25%)	73 (41%)	37 (29%)	325 (32%)
2		81 (23%)	9 (8%)	26 (11%)	15 (18%)	8 (8%)	46 (25%)	12 (7%)	30 (23%)	146 (14%)
3		101 (29%)	9 (8%)	11 (5%)	17 (20%)	5 (5%)	53 (29%)	24 (14%)	33 (26%)	152 (15%)
4		28 (8%)	1 (1%)	0	19 (23%)	0	9 (5%)	5 (3%)	13 (10%)	47 (5%)
**Ballooning grade**										
0		78 (22%)	64 (58%)	127 (52%)	35 (42%)	28 (27%)	44 (24%)	58 (33%)	5 (4%)	361 (35%)
1		142 (41%)	35 (32%)	92 (38%)	39 (47%)	63 (61%)	73 (40%)	78 (44%)	64 (50%)	444 (43%)
2		130 (37%)	11 (10%)	23 (10%)	9 (11%)	13 (12%)	65 (36%)	40 (23%)	60 (47%)	221 (22%)
**Lobular inflammation grade**										
0		72 (21%)	71 (65%)	110 (45%)	0	18 (17%)	38 (21%)	3 (2%)	2 (2%)	242 (24%)
1		224 (64%)	33 (30%)	111 (46%)	35 (42%)	66 (63%)	123 (68%)	100 (57%)	51 (40%)	519 (51%)
2		50 (14%)	5 (5%)	20 (8%)	45 (54%)	17 (16%)	21 (12%)	67 (38%)	49 (38%)	224 (22%)
3		4 (1%)	1 (1%)	1 (<1%)	3 (4%)	3 (3%)	0	6 (3%)	27 (21%)	41 (4%)
**Steatosis grade**										
0		17 (5%)	37 (34%)	56 (23%)	0	0	12 (7%)	4 (2%)	0	109 (11%)
1		87 (25%)	27 (25%)	90 (37%)	34 (41%)	44 (42%)	81 (45%)	48 (27%)	18 (14%)	342 (33%)
2		108 (31%)	21 (19%)	56 (23%)	30 (36%)	43 (41%)	48 (26%)	92 (52%)	46 (36%)	336 (33%)
3		138 (39%)	25 (23%)	40 (17%)	19 (23%)	17 (16%)	41 (23%)	32 (18%)	65 (50%)	239 (23%)
NAS score ≥4		239 (68%)	36 (33%)	81 (33%)	50 (60%)	47 (45%)	110 (60%)	115 (65%)	120 (93%)	559 (54%)
NASH		241 (69%)	31 (28%)	92 (38%)	48 (58%)	63 (61%)	122 (67%)	116 (66%)	123 (95%)	595 (58%)
NASH + NAS ≥ 4 + F ≥ 2		174 (50%)	16 (15%)	28 (12%)	36 (43%)	9 (9%)	78 (43%)	36 (20%)	74 (57%)	277 (27%)
**Time between procedures (days)**										
Time between FibroScan and liver biopsy, median (IQR); range		0 (0 to 0); −14 to 12	78 (49 to 162); −328 to 332	56 (40 to 84); −33 to 309	1 (−2 to 1); −95 to 161	0 (0 to 0); −84 to 9	0 (0 to 0); 0 to 0	0 (0 to 0); 0 to 0	35 (16 to 113); −271 to 360	1 (0 to 55); −328 to 360
Time between FibroScan and blood analyses, median (IQR); range		0 (0 to 0); −1 to 9	..	9 (0 to 27); −151 to 217	0 (0 to 0); 0 to 0	0 (0 to 0); −84 to 9	0 (0 to 0); 0 to 0	0 (0 to 0); 0 to 0	18 (1 to 113); −315 to 373	0 (0 to 4); −315 to 373
Time between liver biopsy and blood analyses, median (IQR); range		0 (0 to 0); −15 to 12	..	−46 (−70 to 22); −309 to 93	−1 (−1 to 2); −161 to 95	0 (0 to 0); 0 to 0	0 (0 to 0); 0 to 0	0 (0 to 0); 0 to 0	−12 (−31 to 36); −435 to 293	0 (−24 to 0); −435 to 293

Data are n, median (IQR), n (%), or mean (SD), unless otherwise specified. The NAS and Kleiner scoring system are described in the appendix (p 2). Data are missing from the French cohort because the investigator did not agree to share those data. In Malaysia, the BMI criteria for obesity are lower than in Western countries. For the Chinese and Malaysian cohorts the median (IQR), minimum and maximum delay were frequently zero because procedures were systematically done on the same day. ALT=alanine aminotransferase. AST=aspartate aminotransferase. BMI=body-mass index. CAP=controlled attenuation parameter. FIB-4=fibrosis-4 index. GGT=γ-glutamyl transferase. LSM=liver stiffness measurement. NAFLD=non-alcoholic fatty liver disease. NASH=non-alcoholic steatohepatitis. NAS=NAFLD activity score. NFS=NAFLD fibrosis score. VCTE=vibration-controlled transient elastography.

Models combining LSM by VCTE; CAP; and AST, ALT, or AAR were compared (appendix p 9). AST was determined to be the best parameter to combine with LSM and CAP. Further nested model comparison was done (appendix p 10), which showed that a model combining LSM, CAP, and AST had significantly better predictive properties than models with only one or two of these predictors. This resulted in the following equation for the FAST score:

FAST=e-1·65+1·07×ln(LSM)+2·66*10-8×CAP3-63·3×AST-11+e-1·65+1·07×ln(LSM)+2·66*10-8×CAP3-63·3×AST-1

**Figure 2:**
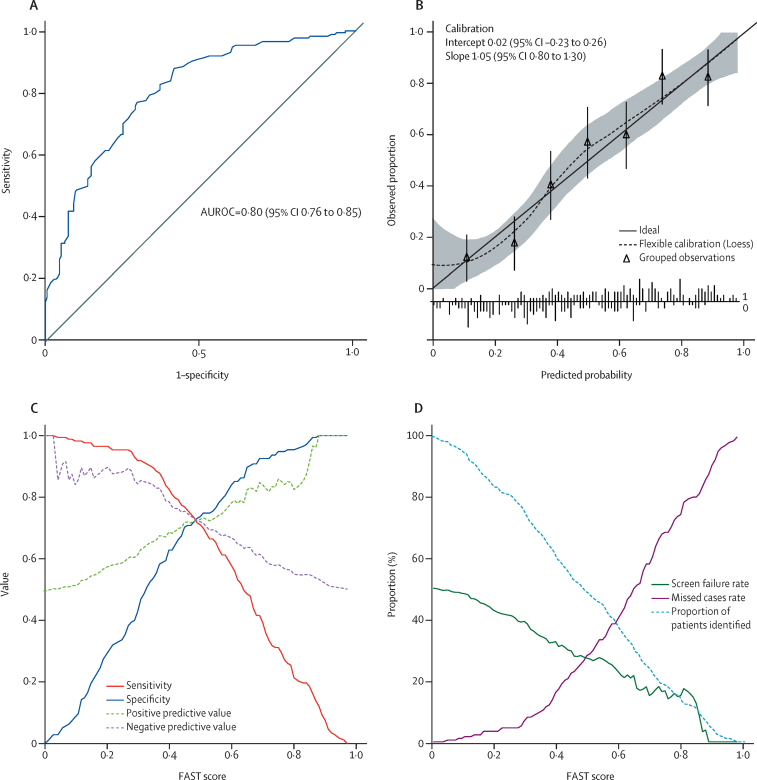
Diagnostic performance in the derivation cohort of the FAST score for the diagnostic of NASH + NAS ≥ 4 + F ≥ 2 (A) Receiver operating characteristic curve. (B) Calibration plot and calibration intercept and slope. The shaded area indicates 95% CI. The calibration plot characterises the agreement between observed proportion and predicted probabilities. The intercept compares the mean of all predicted risks with the mean observed risk and indicates the extent that predictions are systematically too low or too high.^19^ The slope accounts for differences in performance in groups at high or low risk. Calibration of the data is estimated using a smoothed regression line (dotted line) using locally estimated scatterplot smoothing (Loess) that allows inspection of the calibration across the range of predicted values and determination of whether there are segments of the range in which the model is poorly calibrated.^19^ Triangles represent deciles of participants (n=50) grouped by similar predicted risk. Calibration of the score is satisfactory since the intercept is not significantly different from 0, slope is not significantly different from 1, the flexible calibration curve is close to the ideal calibration (solid line), and its CI zone includes the ideal curve. (C) Sensitivity, specificity, positive predictive value, and negative predictive value versus all possible FAST score values. (D) Screen failure rate, missed cases rate, and proportion of patients identified, versus FAST scores values. Plot of the screen failure rate (equal to 1–positive predictive value) and missed cases rate (equal to 1–sensitivity) versus all possible FAST score values. At given FAST score cutoffs, it is possible to graphically assess the screen failure rate and missed cases rate together with the proportion of patients above the FAST score who would be given liver biopsy in the context of patients screening in drug trials for NASH. NASH=non-alcoholic steatohepatitis. FAST=FibroScan-aspartate aminotransferase. NASH + NAS ≥ 4 + F ≥ 2=NASH, elevated non-alcoholic fatty liver disease activity score (≥4), and advanced fibrosis (≥stage 2). AUROC=area under the receiver operating curve.

The FAST score was sensitive to each individual histological component (appendix p 20). As the derived FAST score is the predicted probability from the logistic regression model it is bounded between 0 and 1, and can be interpreted in a probabilistic manner. AUROC in the bootstrap sample (apparent performance) was 0·803 (95% CI 0·758–0·849) and in the original data (test performance) 0·804 (0·790–0·806), showing little over-optimism (−6·0 × 10^−4^, −4·3 × 10^−2^ to 4·7 × 10^−2^). Predictive performance of FAST score in terms of discrimination, calibration, and diagnostic accuracy (figure 2) indicated an AUROC of 0·80 (95% CI 0·76–0·85) with satisfactory calibration of predicted probabilities.

Diagnostic performance of FAST score in terms of sensitivity, specificity, and positive and negative predictive values is represented in figure 2C by cutoff value. Figure 2D illustrates the performance of FAST score as it might be used in the context of identifying patients for therapies or drug trials for NASH. The screen failure rate represents the proportion of screened participants having liver biopsy that would not meet the histological target (NASH + NAS ≥ 4 + F ≥ 2) and would therefore not be randomised in trials or considered for treatment. If FAST score was used to identify such patients, the screen failure rate would decrease from 174 (50%) of 350 patients with increasing FAST cutoffs as illustrated in figure 2D, although more patients would be identified as false negatives for NASH + NAS ≥ 4 + F ≥ 2 (missed case rate). The histological characteristics of misclassified patients are detailed in the appendix (p 19).

**Figure 3:**
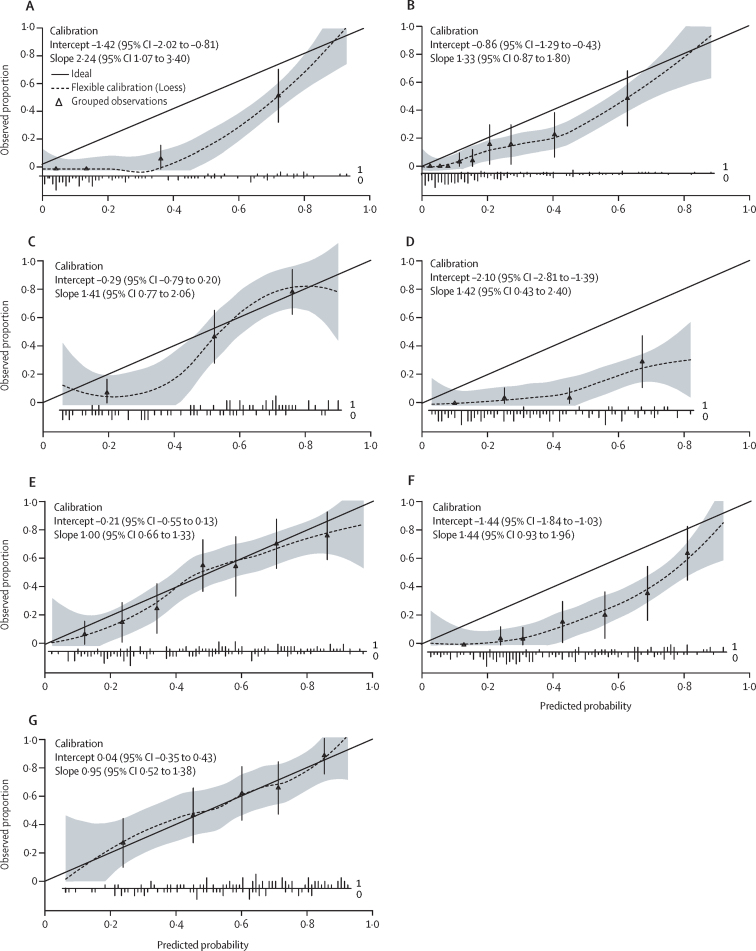
Calibration plots in external validation cohorts (A) French bariatric cohort (n=110). Prevalence of NASH + NAS ≥ 4 + F ≥ 2=15%. (B) USA screening cohort (n=242). Prevalence of NASH + NAS ≥ 4 + F ≥ 2=12%. (C) China Hong-Kong NAFLD cohort (n=83). Prevalence of NASH+NAS≥4+F≥2=43%. (D) China Wenzhou NAFLD cohort (n=104). Prevalence of NASH + NAS ≥ 4 + F ≥ 2=9%. (E) French NAFLD cohort (n=182). Prevalence of NASH + NAS ≥ 4 + F ≥ 2=43%. (F) Malaysian NAFLD cohort (n=176). Prevalence of NASH + NAS ≥ 4 + F ≥ 2=20%. (G) Turkish NAFLD cohort (n=129). Prevalence of NASH + NAS ≥ 4 + F ≥ 2=57%. The solid line in each image represents the ideal calibration. The dotted line represents the calibrations estimated on the data using locally estimated scatterplot smoothing (Loess). The shaded area indicates 95% CI. Triangles represent deciles of participants grouped by similar predicted risk. The distribution of participants is indicated with spikes at the bottom of the graph (patients with NASH + NAS ≥ 4 + F ≥ 2 above the x-axis, patients without NASH + NAS ≥ 4 + F ≥ 2 below the x-axis). The French (E) and Turkish (G) NAFLD external validation cohorts are well calibrated; their calibration curve is nearly linear, their intercept is close to zero (CIs include zero), and their slope is close to one (CIs include one). The Chinese Hong-Kong NAFLD cohort (C) has a zone in which the risk of being NASH + NAS ≥ 4 + F ≥ 2 is overestimated using the FAST score (grey ribbon below the ideal calibration curve) and a zone in which the calibration seem adequate (grey ribbon zone includes the ideal calibration curve). However, this cohort size is quite small (n=83). The French bariatric surgery (A), USA screening (B), Chinese Wenzhou NALFD (D), and the Malaysian NAFLD (F) cohort have a range of prevalence of NASH + NAS ≥ 4 + F ≥ 2 (9% to 20%), which is lower than the derivation cohort. In those four cohorts, the FAST score overestimates the probability of being NASH + NAS ≥ 4 + F ≥ 2. The discrepancy is mainly driven by the intercept (CIs do not include zero). All slopes are within an acceptable range (the CI includes one), except for the French bariatric cohort, which seems to be at the limit. NAFLD=non-alcoholic fatty liver disease. FAST=FibroScan-aspartate aminotransferase. NASH + NAS ≥ 4 + F ≥ 2=non-alcoholic steatohepatitis, elevated non-alcoholic fatty liver disease activity score (≥4) and advanced fibrosis (≥stage 2).

External validation of the score was evaluated alongside calibration plots for each external validation cohort (figure 3). Calibration was satisfactory for the Chinese Hong-Kong, French, and Turkish NAFLD cohorts, which have a prevalence of NASH + NAS ≥ 4 + F ≥ 2 similar to the derivation cohort. However, for cohorts with a lower prevalence of the outcome, FAST score overestimates the probability of having NASH + NAS ≥ 4 + F ≥ 2. Corresponding AUROCs are provided (table 2), with good to excellent discrimination in all external validation cohorts except for the Turkish NAFLD cohort, which had modest performance. Best discrimination was observed in the French bariatric surgery cohort, with an AUROC of 0·95 (95% CI 0·91–0·99).

**Table 2:** Diagnostic performance of the FAST score for the diagnosis of NASH + NAS ≥ 4 + F ≥ 2 in the derivation and external validation cohorts

	**AUROC (95% CI)**	**n**	**Prevalence of NASH + NAS ≥ 4 + F ≥ 2**	**Rule-out zone (FAST ≤0·35)**				**Grey zone (FAST 0·35–0·67), n (%)**	**Rule-in zone (FAST ≥0·67)**			
				n (%)	Sensitivity	Specificity	NPV		n (%)	Specificity	Sensitivity	PPV
Derivation cohort	0·80 (0·76–0·85)	350	174 (50%)	113 (32%)	0·90 (157/174)	0·53 (93/176)	0·85 (93/110)	136 (39%)	101 (29%)	0·90 (159/176)	0·48 (84/174)	0·83 (84/101)
French bariatric surgery cohort	0·95 (0·91–0·99)	110	16 (15%)	69 (63%)	1·00 (16/16)	0·73 (69/94)	1·00 (69/69)	22 (20%)	19 (17%)	0·93 (87/94)	0·75 (12/16)	0·63 (12/19)
USA screening cohort	0·86 (0·80–0·93)	242	28 (12%)	194 (80%)	0·64 (18/28)	0·86 (183/214)	0·95 (183/193)	39 (16%)	9 (4%)	0·99 (212/214)	0·25 (7/28)	0·78 (7/9)
China Hong-Kong NAFLD cohort	0·85 (0·76–0·93)	83	36 (43%)	28 (34%)	0·94 (34/36)	0·55 (26/47)	0·93 (26/28)	29 (35%)	26 (31%)	0·89 (42/47)	0·58 (21/36)	0·81 (21/26)
China Wenzhou NAFLD cohort	0·84 (0·73–0·95)	104	9 (9%)	55 (53%)	0·89 (8/9)	0·56 (53/95)	0·98 (58/67)	37 (36%)	12 (11%)	0·92 (87/95)	0·44 (4/9)	0·33 (4/12)
French NAFLD cohort	0·80 (0·73–0·86)	182	78 (43%)	67 (37%)	0·88 (69/78)	0·56 (58/104)	0·87 (58/67)	69 (38%)	46 (24%)	0·89 (93/104)	0·45 (35/78)	0·76 (35/46)
Malaysian NAFLD cohort	0·85 (0·78–0·91)	176	36 (20%)	78 (44%)	0·94 (34/36)	0·54 (75/140)	0·97 (75/77)	59 (34%)	39 (22%)	0·87 (122/140)	0·58 (21/36)	0·54 (21/39)
Turkish NAFLD cohort	0·74 (0·65–0·82)	129	74 (57%)	26 (20%)	0·91 (67/74)	0·35 (19/55)	0·73 (19/26)	57 (44%)	46 (36%)	0·82 (45/55)	0·49 (36/74)	0·78 (36/46)
Pooled external patients cohort	0·85 (0·83–0·87)	1026	277 (27%)	517 (51%)	0·89 (246/277)	0·64 (483/749)	0·94 (483/514)	312 (30%)	197 (19%)	0·92 (688/749)	0·49 (136/277)	0·69 (136/197)

Performance associated with dual cutoff approach is evaluated using the FAST score when the cutoffs are calculated in the derivation cohort and applied in all external validation cohorts. The lower cutoff constitutes a rule-out cutoff and is based on a sensitivity ≥0·90 in the derivation cohort. The higher cutoff constitutes a rule-in cutoff and is based on a specificity ≥0·90 in the derivation cohort. Individuals with a FAST score in between the rule-out and rule-in cutoffs are in the grey zone. In the rule-out group, the sensitivity is provided together with the specificity and NPV to appraise the rule-out performance of the score. In the rule-in group, the specificity is provided together with the sensitivity and PPV to appraise the rule-in performance of the score. AUROC=area under the receiver operating curve. FAST=FibroScan-aspartate aminotransferase. NAFLD=non-alcoholic fatty liver disease. NASH=non-alcoholic steatohepatitis. NAS=NAFLD activity score. NASH + NAS ≥ 4 + F ≥ 2=NASH and NAS≥4 and advanced fibrosis (≥stage 2). NPV=negative predictive value. PPV=positive predictive value.

Cutoff for sensitivity (≥0·90) was 0·35 and for specificity (≥0·90) was 0·67 in the derivation cohort (full diagnostic performance in the appendix, p 11), with characteristics for validation cohorts detailed in table 2. Using the dual cutoff approach, PPV in the derivation cohort was 0·83 (84/[84+17], 95% CI 0·75–0·87), NPV was 0·85 (93/[93+17], 0·77–0·88), and 136 (39%) of 350 patients were in the grey zone between the two cutoffs. When applying these cutoffs to the external validation cohorts, PPV was in the same order of magnitude with a similar sensitivity in the Chinese Hong-Kong, French, and Turkish NAFLD cohorts. The USA screening cohort had a PPV in the same order of magnitude but a lower sensitivity. The Chinese Wenzhou NAFLD with the lowest prevalence of NASH + NAS ≥ 4 + F ≥ 2, had a lower PPV but similar sensitivity. NPV was high in all external validation cohorts.

FAST was compared with FIB-4 and the NAFLD fibrosis score (NFS) for the identification of patients with NASH + NAS ≥ 4 + F ≥ 2 in the subgroup of patients from the derivation and external validation cohorts that had all parameters needed to compute those three scores (derivation cohort n=339, pooled external validation cohort n=981). Corresponding AUROCs and diagnostic performance using the dual cutoff approach (appendix p 12) were inferior to the FAST score. Indeed, discrimination was significantly higher for the FAST score in the derivation and in the pooled external validation cohort. Using the dual cutoff approach in the pooled validation cohort, NASH + NAS ≥ 4 + F ≥ 2 yielded a similar number of patients in the grey zone (311 [32%] of 981 patients) as did FAST (304 [31%]), NASH + NAS ≥ 4 + F ≥ 2 yielded a slightly higher PPV (0·72 [18/25] with NASH + NAS ≥ 4 + F ≥ 2 *vs* 0·69 [132/190] with FAST), but lower NPV (0·83 [536/645] with NASH + NAS ≥ 4 + F ≥ 2 *vs* 0·94 [453/484] with FAST), and failed to identify most of the patients with NASH+NAS≥4+F≥2 (sensitivity 0·07 [18/272] with NASH + NAS ≥ 4 + F ≥ 2 *vs* 0·49 [132/272] with FAST). NFS had a larger grey zone than FAST (444 [45%] of 981 patients with NFS *vs* 304 [31%] of 981 patients with FAST) with lower PPV (NFS 0·50 [51/101] *vs* FAST 0·69 [132/190]) and NPV (NFS 0·85 [370/436] *vs* FAST 0·94 [453/484]). Moreover, the addition of metabolic parameters to the score were appraised and did not provide significant improvement in terms of discrimination (appendix p 15).

## Discussion

In this prospective cohort study, we present a new, simple, non-proprietary score that identifies patients with progressive NASH and has been validated in multiple large global cohorts.

There has been considerable debate as to which patients with NASH should be the focus of monitoring and treatment, although data have suggested that the degree of fibrosis is a major driver of clinical outcomes.^22^, ^23^, ^24^ Our choice of NASH with NAS ≥ 4 and F ≥ 2 is based on this literature and also many therapeutic studies that show the presence of elevated necro-inflammatory activity is linked to progressive injury and pharmacological response.^12^, ^25^

The FAST score, in keeping with recommended practice, was configured to have two thresholds, a rule-out and a rule-in cutoff. This allowed for classification of more than 70% of patients in the validation cohorts. Moreover, FAST score has good performance characteristics with a negative likelihood ratio of 0·2 (rule-out, cutoff) and a positive likelihood ratio of 5 (rule-in, cutoff), ratios that are maintained in the validation groups. Thus, this test could have a substantial influence on clinical decision making and be an important adjunct in identifying patients for clinical trials or commencement of pharmacotherapies. 33 patients had types of lesion on their biopsy samples that were neither in-keeping with NAFLD nor were normal. Although some of these patients had some fibrosis or steatosis, the pathologist did not feel it was appropriate to grade them for items of the NAS or fibrosis according to the NASH CRN scoring system, which would have biased the construction of the score. We therefore decided to build the score using a completely NAFLD cohort. Although this might affect the performance of the score in the derivation cohort, it would have no effect on performance in the multiple validation cohorts.

As with any other predictive models, the performance of the FAST score will be determined by the population to which it is applied: the spectrum bias will affect the sensitivity and specificity of the score, and the prevalence of the target patients will affect predictive values.^26^ For example, the PPV of any test will be different in primary versus secondary care, although the NPV will remain robust. This test will be of greatest value in secondary care, in which consideration for biopsy and eligibility for trials or treatment will be evaluated. These patients will inevitably have received some form of vetting, but we know from screening data for clinical trials that there is a high rate of screen failure for trials. We believe the FAST score will help to identify the patients suitable for clinical trials and emerging therapies more efficiently and reduce unnecessary liver biopsies.

Using such tools is inevitably a trade-off between reducing false positives and avoiding too many false negatives. This requires an understanding of the relative impact of these effects (ie, fewer biopsies and the potential problems that result from patients not being considered for treatment), which is limited, and the efficacy of new treatments is unknown.

In generating this score, we sought to determine the performance of standard liver blood tests or other widely used algorithms in identifying patients with progressive NASH. The performance of these parameters in isolation was inferior to the FAST score (appendix p 9), and indeed other than the addition of AST values, there was no evidence that addition of other elements (eg, metabolic syndrome parameters) improved the performance of the FAST score (appendix p 15).

One of the challenges with such tests is dealing with patients in the grey zone. Decision making for such patients will be influenced by individual characteristics, proximity to thresholds (low or high), and operator confidence. In many cases, it might be appropriate to repeat the test at a suitable interval (eg, 2–3 months), whereas in others it might be useful to consider an alternative modality or proceed with liver biopsy. Patient choice will be a key determinant of this decision making.

We, and others, have shown that CAP and LSM by VCTE measurements are widely applicable in patients with NASH, with a low failure rate (3%) and good performance in determining the degree of liver steatosis and fibrosis.^11^, ^27^ Importantly, we also showed that LSM values only correlated with fibrosis, and were not influenced by other histological parameters or type of probe used.

Our study has several strengths. Firstly, it is prospective in nature and has undergone extensive validation across multiple large global cohorts. Secondly, FAST score performance is good across the full range of validation cohorts. AUROC ranged from 0·74 to 0·95, with PPV up to 0·85 and NPV ranging from 0·73 to 1 using the dual cutoffs approach, with cutoffs derived in the derivation cohort. Thirdly, the wide availability of FibroScan devices based on VCTE technology, the need for just a serum AST value, its non-invasive nature, its low cost per scan, and its modest requirement to attain technical proficiency required to do the scans, mean the method can be rolled out easily across most clinical practices. Roll-out is further aided by the free availability of the equation, which is also accessible through an app.

There are several weaknesses to this study, including the requirement for a FibroScan device, which could affect uptake. Use of FAST in primary care will require investment in devices and personnel, although there are many examples of such models being introduced successfully.^6^ Another potential criticism is that our score focuses on patients with fibrosis stage 2 or higher, with some contending that identifying patients with more advanced fibrosis (≥stage 3) should be the priority. At this stage, clinical trials are aiming to recruit patients with stage 2 and 3 fibrosis, and we therefore believe that stage 2 fibrosis and above constitutes a reasonable target group. We derived cutoffs for the identification of patients with advanced fibrosis (F≥3) in our cohort (appendix p 16,17). The performance characteristics were good, with moderate likelihood ratios to rule-out and rule-in such patients.

In two validation cohorts there was only access to the M probe, so patients with a BMI greater than 32 kg/m^2^ were excluded, which could bias the performance of the score in those cohorts.

Finally, FAST score performed least well in terms of calibration in low prevalence populations, and caution should be exercised when interpreting the score in these settings, although discrimination performance of the score was good. In a future study, recalibration of scores could be considered to correct miscalibration while keeping the same level of discrimination. However, to do so we need to know which prevalence would be representative of the population, in whom the score would be used, and a robust reference cohort with that low prevalence.

In summary, we believe the FAST score will allow for the more efficient identification of an at-risk group of patients with progressive NASH that merit consideration for further treatment.

**This online publication has been corrected. The corrected version first appeared at thelancet.com/gastrohep on March 11, 2020**
