# Correction to *Lancet Gastroenterol Hepatol* 2020; 5: 362–73

**DOI:** 10.1016/S2468-1253(20)30055-8

**Published:** 2020-03-11

*Newsome PN, Sasso M, Deeks JJ, et al. FibroScan-AST (FAST) score for the non-invasive identification of patients with non-alcoholic steatohepatitis with significant activity and fibrosis: a prospective derivation and global validation study.* Lancet Gastroenterol Hepatol *2020; **5:** 362–73—*In table 2 of this Article, the title of the second column under the heading “Rule-in zone (FAST ≥0·67)” should read “Specificity” and the title of the third column “Sensitivity”. This correction has been made online as of March 11, 2020 and the printed Article is correct.

