# Supplementary material for: FibroScan-AST (FAST) score for the non-invasive identification of patients with non-alcoholic steatohepatitis with significant activity and fibrosis: a prospective derivation and global validation study
Source: Lancet Gastroenterol Hepatol. 2020 Feb 3;5(4):362–73. doi: 10.1016/S2468-1253(19)30383-8 (PMC7066580; doi:10.1016/S2468-1253(19)30383-8)
Supplement: Supplementary appendix [file mmc1.pdf]

THE LANCET
Gastroenterology & Hepatology

Supplementary appendix

This appendix formed part of the original submission and has been peer reviewed. We post it as supplied by the authors.

Supplement to: Newsome PN, Sasso M, Deeks JJ, et al. FibroScan-AST (FAST) score for the non-invasive identification of patients with non-alcoholic steatohepatitis with significant activity and fibrosis: a prospective derivation and global validation study. *Lancet Gastroenterol Hepatol* 2020; published online Feb 3. https://doi.org/10.1016/S2468-1253(19)30383-8.

SUPPLEMENTARY MATERIAL

Supplementary Methods 2

Supplementary Table 1 – Transparent reporting of a multivariable prediction model for individual prognosis or diagnosis TRIPOD checklist 3

Supplementary Table 2 – External validation cohorts description 5

Supplementary Table 3 – Potential risk of bias in each external derivation cohort 7

Supplementary Table 4 – Comparison of model combining AST, ALT or AAR with CAP and LSM by VCTE 9

Supplementary Table 5 – Comparison of all possible nested models 10

Supplementary Table 6 – FAST cut-offs in the derivation cohort for a sensitivity ≥ 0·90 and a specificity ≥ 0·90 and associated diagnostic performance 11

Supplementary Table 7 – Performance comparison of the FAST score with FIB-4 and NFS using the dual-cut-off approach (0·35 and 0·67 for FAST, 1·30 and 3·25 for FIB-4 and -1·455 and 0·676 for NFS), in the derivation and external validation cohorts 12

Supplementary Table 8 – Comparison of diagnostic performance of the FAST score and the score combining LSM by VCTE, CAP, AST and DM or HTN 15

Supplementary Table 9 – FAST cut-offs for a sensitivity ≥ 0·90 and a specificity ≥ 0·90 and associated diagnostic performance, for the identification of patients with NASH+NAS≥4+F≥3 16

Supplementary Table 10: Diagnostic performance of the FAST score for the diagnostic of NASH+NAS≥4+F≥3 in the derivation and external validation cohorts 17

Supplementary Table 11: Comparison of FAST score, FAST score individual parameters and histological parameters between correctly classified and misclassified patients in the rule-out and rule-in zones 19

Supplementary Figure 1. Boxplot of FAST score in the derivation cohort versus (A) fibrosis stage, (B) steatosis grade, (C) lobular inflammation grade, (D) ballooning grade. 20

References 21

Supplementary Methods

Histopathologic evaluation

Steatosis (from 0 to 3), ballooning (from 0 to 2), lobular inflammation (from 0 to 3), fibrosis (from 0 to 4) and NAFLD activity score (NAS) were scored using the NASH clinical research network (NASH CRN) scoring system.[1] NAS score was the sum of steatosis, ballooning and lobular inflammation grades and ranged from 0 to 8.[1] NASH was diagnosed using the "fatty liver: inhibition of progression" (FLIP) definition as the presence of steatosis, hepatocyte ballooning and lobular inflammation with at least 1 point for each category.[2]

Supplementary Table 1 – Transparent reporting of a multivariable prediction model for individual prognosis or diagnosis TRIPOD checklist

Section/Topic			Checklist Item	Page
**Title and abstract**				
Title	1	D;V	Identify the study as developing and/or validating a multivariable prediction model, the target population, and the outcome to be predicted.	1
Abstract	2	D;V	Provide a summary of objectives, study design, setting, participants, sample size, predictors, outcome, statistical analysis, results, and conclusions.	7-8
**Introduction**				
Background and objectives	3a	D;V	Explain the medical context (including whether diagnostic or prognostic) and rationale for developing or validating the multivariable prediction model, including references to existing models.	9
	3b	D;V	Specify the objectives, including whether the study describes the development or validation of the model or both.	9-10
**Methods**				
Source of data	4a	D;V	Describe the study design or source of data (e.g., randomized trial, cohort, or registry data), separately for the development and validation data sets, if applicable.	D: 10<br>V: 13-14, Supp Table 2
	4b	D;V	Specify the key study dates, including start of accrual; end of accrual; and, if applicable, end of follow-up.	D: 10<br>V: Supp Table 2
Participants	5a	D;V	Specify key elements of the study setting (e.g., primary care, secondary care, general population) including number and location of centres.	D: 10<br>V: Supp Table 2
	5b	D;V	Describe eligibility criteria for participants.	D:11<br>V: 14
	5c	D;V	Give details of treatments received, if relevant.	Not applicable
Outcome	6a	D;V	Clearly define the outcome that is predicted by the prediction model, including how and when assessed.	D:11, 13<br>V: 11, 14, Supp Table 2
	6b	D;V	Report any actions to blind assessment of the outcome to be predicted.	D: 11<br>V: 13
Predictors	7a	D;V	Clearly define all predictors used in developing or validating the multivariable prediction model, including how and when they were measured.	D: 11, 13<br>V: 13
	7b	D;V	Report any actions to blind assessment of predictors for the outcome and other predictors.	D:12<br>V: 13
Sample size	8	D;V	Explain how the study size was arrived at.	12
Missing data	9	D;V	Describe how missing data were handled (e.g., complete-case analysis, single imputation, multiple imputation) with details of any imputation method.	14
Statistical analysis methods	10a	D	Describe how predictors were handled in the analyses.	14
	10b	D	Specify type of model, all model-building procedures (including any predictor selection), and method for internal validation.	14
	10c	V	For validation, describe how the predictions were calculated.	15
	10d	D;V	Specify all measures used to assess model performance and, if relevant, to compare multiple models.	15
	10e	V	Describe any model updating (e.g., recalibration) arising from the validation, if done.	Not applicable
Risk groups	11	D;V	Provide details on how risk groups were created, if done.	Not applicable
Development vs. validation	12	V	For validation, identify any differences from the development data in setting, eligibility criteria, outcome, and predictors.	Supp Table 3
**Results**				
Participants	13a	D;V	Describe the flow of participants through the study, including the number of participants with and without the outcome and, if applicable, a summary of the follow-up time. A diagram may be helpful.	Figure 1, Supp Table 2
	13b	D;V	Describe the characteristics of the participants (basic demographics, clinical features, available predictors), including the number of participants with missing data for predictors and outcome.	Table 1
	13c	V	For validation, show a comparison with the development data of the distribution of important variables (demographics, predictors and outcome).	Table 1
Model development	14a	D	Specify the number of participants and outcome events in each analysis.	Table 1
	14b	D	If done, report the unadjusted association between each candidate predictor and outcome.	Supp Table 4<br>Supp Table 5<br>Supp Table 8
Model specification	15a	D	Present the full prediction model to allow predictions for individuals (i.e., all regression coefficients, and model intercept or baseline survival at a given time point).	17
	15b	D	Explain how to the use the prediction model.	17
Model performance	16	D;V	Report performance measures (with CIs) for the prediction model.	D: 17-19, Table 2, Figure 2<br>V: 18-19, Table 2, Figure 3
Model-updating	17	V	If done, report the results from any model updating (i.e., model specification, model performance).	Not applicable

**Discussion**				
Limitations	18	D;V	Discuss any limitations of the study (such as non-representative sample, few events per predictor, missing data).	21
Interpretation	19a	V	For validation, discuss the results with reference to performance in the development data, and any other validation data.	20-21
	19b	D;V	Give an overall interpretation of the results, considering objectives, limitations, results from similar studies, and other relevant evidence.	20-21
Implications	20	D;V	Discuss the potential clinical use of the model and implications for future research.	21-22
**Other information**				
Supplementary information	21	D;V	Provide information about the availability of supplementary resources, such as study protocol, Web calculator, and data sets.	21
Funding	22	D;V	Give the source of funding and the role of the funders for the present study.	16

Supplementary Table 2 – External validation cohorts description

		French bariatric surgery cohort	USA screening cohort	China Hong-Kong NAFLD cohort	China Wenzhou NAFLD cohort	French NAFLD cohort	Malaysian NAFLD cohort	Turkish NAFLD cohort
**Study description**	**Funding**	Unrestricted grant from Echosens and Novo Nordisk	Echosens	Grant from the Research grant Council of Hong-Kong government	Training funding by the High level creative Talents from Department of public health in Zhejiang province	No funding	Research grant from the university of Malaya	Scientific research fund from Marmara university
	**Enrolment dates (first and last inclusion)**	From 2012/04 to 2015/05	From 2015/08 to 2018/07	From 2003/05 to 2017/11	From 2017/01 to 2018/03	From 2013/06 to 2018/06	From 2012/11 to 2015/10	From 2016/01 to 2018/09
	**Study design**	Prospective cross-sectional single centre study	Prospective cross-sectional single centre study	Prospective cross-sectional single centre study	Prospective cross-sectional single centre study·	Prospective cross-sectional single centre study	Prospective cross-sectional single centre study	Prospective cross-sectional single centre study
	**PMID if data were used for publication**	NA	NA	PMID-30658987<br>PMID-28506907<br>PMID-23032979<br>PMID-2010754	NA	PMID-29577364	PMID-24548002<br>PMID-25788185<br>PMID-25184298	NA
	**Center description**	Bariatric surgery centre	Tertiary military medical Centre	Hepatology tertiary care	Hepatology tertiary care	Hepatology tertiary care	Hepatology tertiary care	Hepatology tertiary care
	**Eligibility criteria**	Inclusion: Severe obese patients' candidate to bariatric surgery with no history of liver disease; liver biopsy performed during the surgery; a LSM performed with VCTE prior to bariatric surgery.<br>Exclusion: current excessive drinking (average daily consumption of > 20 g alcohol/day for women and > 30 g alcohol/day for men); long-term consumption of	Inclusion/exclusion: Males and females, >18 and <80 years of age without a known history of NAFLD or other chronic liver disease to include HCV, HBV; alcohol ingestion greater than the accepted range for NAFLD (men >21 per week, women > 14 per week) and chronic use of steatogenic medications (amiodarone, methotrexate, etc) were considered exclusionary	Inclusion: age > 18 years; biopsy-proven NAFLD· Exclusion: other liver disease; excessive alcohol consumption; secondary fatty liver (e·g· DILI); history of liver surgery or liver transplantation; history of HCC; history of malignancy unless if complete remission > 5 years	Inclusion: age 18-75 years; BMI < 35 kg/m^2; US, CT or MRI imaging showing fatty liver disease; abnormal ALT but below 5 ULN; no alcohol drinking history or daily alcohol intake < 20 g for male and 10 g for female	Inclusion: LB scheduled of the evaluation of NAFLD	Inclusion: NAFLD patients diagnosed on US following exclusion of other cause of CLD including alcohol	Inclusion: evidence of hepatic steatosis on US; abnormal liver enzymes or organomegaly; absence of secondary causes of hepatic fat accumulation (e.g. significant alcohol consumption and previous use of steatogenic drugs)·<br>Exclusion: patients with viral hepatitis, DILI, autoimmune hepatitis, metabolic/genetic liver disease or low platelets count (< 100 x 10^9/L), history of

		hepatotoxic drugs; viral hepatitis, haemochromatosis						malignancy and heart failure
**FibroScan device information**	**Probe used**	Both M and XL	Both M and XL	Both M and XL	M only	Both M and XL	M only	Both M and XL
	**Probe selection**	Automatic probe selection tool	Automatic probe selection tool	Both probes on all patients	NA	Both probes on all patients	NA	Automatic probe selection tool
	**Number of FibroScan operators and experience**	N=2<br>Novice	N=2<br>Over 1 year experience for both	N=4<br>Experience >100 VCTE examinations for all	N=1<br>5 years' experience	N=3<br>Experience >1000 VCTE examinations for all	N=2<br>Experience >200 VCTE examinations for both	N=1<br>Experience >10000 VCTE examinations
**Histological information**	**Reason to send a patient to LB**	Bariatric surgery	LSM by VCTE, MRE, MRI-PDFF or cT1 above predefined cut-offs	Persistent elevated transaminase, high metabolic burden suspicious of advanced disease, elevated LSM by VCTE	Persistent elevated transaminase or elevated LSM by VCTE or CAP (especially LSM)	Abnormal liver function tests, hyperferritinaemia, metabolic syndrome, abnormal non-invasive tests of liver fibrosis (Fib4, NFS, FibroMeter, LSM by VCTE)	Persistent ALT or AST ≥ 40 or reasons for NASH to be suspected (e·g· significant fibrosis at LSM by VCTE, obese patient with metabolic syndrome)	Evidence of hepatic steatosis on US, abnormal liver enzymes or organomegaly, absence of secondary causes of hepatic fat accumulation (e·g· significant alcohol consumption and previous use of steatogenic drugs), LSM by VCTE > 6 kPa or rarely patients with LSM by VCTE < 6 kPa to exclude other CLD
	**LB reading**	Central double blind reading with consensus by 2 experts pathologists	Central double blind reading with consensus by 2 experts pathologists	Central reading by a single expert pathologist	Routine reading by a single expert pathologist	Central reading by a single expert pathologist	Central reading by a single expert pathologist	Central reading by a single expert pathologist

ALT: alanine transaminase, AST: aspartate aminotransferase, BMI: body mass index, CAP: controlled attenuation parameter, CLD: chronic liver disease, CT: computed tomography, cT1: corrected T1, DILI: drug-induced liver injury, Fib4: fibrosis-4 index, HCC: hepatocellular carcinoma, LB: liver biopsy, LSM: liver stiffness measurement, MRE: magnetic resonance elastography, MRI: magnetic resonance imaging, MRI-PDFF: MRI proton density fat fraction, NAFLD: non-alcoholic fatty liver disease, NASH: non-alcoholic steatohepatitis, NFS: NAFLD fibrosis score, PMID: PubMed identifier, ULN: upper limits of normal, US: ultrasound, USA: United States of America, VCTE: vibration-controlled transient elastography.

Supplementary Table 3 – Potential risk of bias in each external derivation cohort

Potential risk of bias was assessed as follows:

- In patient selection a potential risk of bias is considered if patients are selected for liver biopsy based on the FAST score parameters which are LSM by VCTE, CAP or AST;
- In LB quality a potential risk of bias for the reference standard is considered when liver biopsy specimens are too small ≤ 15 mm. We considered that when more than half of the patients had a LB of sufficient size, the risk of bias was low. For wedge liver biopsies we considered it as unclear since wedge liver biopsy are supposed to be of better quality than needle liver biopsy specimens. Risk of bias was unclear in the case of reading by a single pathologist and low risk in the case of central double-blind reading;
- In FibroScan examination a risk of bias is considered for LSM by VCTE and CAP measurement if automatic probe selection tool was not used. The risk of bias was low when both M and XL probes were available and the probe selection was made automatically by the FibroScan device.
- For the timing, a risk of bias was considered if the median time was greater than two weeks or when there was no information about timing available. When only a few patients had a time interval greater than two weeks, the risk was categorized as unclear.

		French bariatric surgery cohort	USA screening cohort	China Hong-Kong NAFLD cohort	China Wenzhou NAFLD cohort	French NAFLD cohort	Malaysian NAFLD cohort	Turkish NAFLD cohort
**Patients selection**	**Potential bias due to patients selected for LB based on FibroScan results or AST**	☑ (LB in patients with planned bariatric surgery)	? (FibroScan is not the only criteria, AST not criteria)	☒	☒	☒	☒	☒
**LB quality**	**Potential bias in LB quality**	? (45% have a LB length > 15mm but wedge biopsy)	☒ (45% have a LB length > 15mm)	☑ (89% have a LB length > 15mm)	? (no LB length provided)	☑ (93% have a LB length > 15mm)	☒ (45% have a LB length > 15mm)	☑ (97% have a LB length > 15mm)
	**Potential bias in LB reading**	☑ (double-blind central reading)	☑ (double-blind central reading)	? (central reading but single pathologist)	? (central reading but single pathologist)	? (routine reading by a signle pathologist)	? (central reading but single pathologist)	? (central reading but single pathologist)
**FibroScan examination**	**Potential bias due to probe availability**	☑ (M / XL probes available)	☑ (M / XL probes available)	☑ (M / XL probes available)	? (M probe only but BMI <32)	☑ (M / XL probes available)	? (M probe only but BMI<32)	☑ (M / XL probes available)
	**Potential bias due to probe selection**	☑ (automatic probe selection)	☑ (automatic probe selection)	☒ (no automatic probe selection)	☒ (only M probe available)	☒ (no automatic probe selection)	☒ (only M probe available)	☑ (automatic probe selection)

**Timing**	**Potential bias due to time interval between FibroScan and LB**	☒ (median time > 2 weeks)	☒ (median time > 2 weeks)	? (a few patients with time interval > 2 weeks)	☑ (same day for all)	☑ (same day for all)	☑ (same day for all)	☒ (median time > 2 weeks)
	**Potential bias due to time interval between FibroScan and AST evaluation**	☒ (no information about timing)	☒ (large proportion of patients with time interval > 2 weeks)	☑ (same day for all)	? (a few patients with time interval > 2 weeks)	☑ (same day for all)	☑ (same day for all)	☒ (median time > 2 weeks)
	**Potential bias due to time interval between LB and AST evaluation**	☒ (no information about timing)	☒ (median time > 2 weeks)	? (a few patients with time interval > 2 weeks)	☑ (same day for all)	☑ (same day for all)	☑ (same day for all)	☒ (large proportion of patients with time interval > 2 weeks)

☑: low risk, ☒: high risk, ?: unclear

AST: aspartate aminotransferase, LB: liver biopsy, USA: United States of America, NAFLD: non-alcoholic fatty liver disease.

Supplementary Table 4 – Comparison of model combining AST, ALT or AAR with CAP and LSM by VCTE

To select the final model parameters, models combining aspartate aminotransferase (AST): alanine transaminase (ALT) and AST/ALT ratio (AAR) were compared using Akaike information criterion (AIC) and Bayesian information criterion (BIC). The contribution of each variable in each model was appraised using the Wald test. The table shows the corresponding results together with each variable coefficient estimates and 95% confidence intervals.

It can be observed that in each model, both LSM by VCTE and CAP are significant predictors, based on the Wald test for the logistic regression coefficient. Either AST or ALT are also significant predictors when combined with LSM by VCTE and CAP. However, the AAR does not show a significant relationship. The model with the smallest AIC and BIC is the model combining AST to LSM by VCTE and CAP. This model was therefore selected.

		**model<br>LSM by VCTE + CAP + AST**	**model<br>LSM by VCTE + CAP + ALT**	**model<br>LSM by VCTE + CAP + AAR**
**Coefficient estimates (95% CI) and variable importance (Wald test)**	**LSM by VCTE**	β_{LSM}= 7·13 (3·67-11·1) x 10^{-2}<br>p=2.0 x 10^{-4}	β_{LSM}= 8·54 (5·07-12·4) x 10^{-2}<br>p=8 x 10^{-6}	β_{LSM}= 7·50 (4·07-11·4) x 10^{-2}<br>p=8 x 10^{-5}
	**CAP**	β_{CAP}= 9·75 (4·85-15·0) x 10^{-3}<br>p=1.8 x 10^{-4}	β_{CAP}= 8·16 (3·46-13·1) x 10^{-3}<br>p=0·0010	β_{CAP}= 8·60 (3·86-13·6) x 10^{-3}<br>p=6.1 x 10^{-4}
	**AST**	β_{AST}= 3·19 (1·98-4·55) x 10^{-2}<br>p=2 x 10^{-6}	–	–
	**ALT**	–	β_{ALT}= 9·66 (3·55-16·1) x 10^{-3}<br>p=0·0028	–
	**AAR**	–	–	β_{AAR}= 4·58 (-2·89-12·2) x 10^{-1}<br>p=0·23
	**AIC**	408·4	431·1	439·5
	**BIC**	423·8	446·5	454·9

AAR: AST/ALT ratio, AIC: Akaike information criterion, ALT: alanine transaminase, AST: aspartate aminotransferase, BIC: Bayesian information criterion, CAP: controlled attenuation parameter, LSM: liver stiffness measurement, VCTE: vibration-controlled transient elastography·

Supplementary Table 5 – Comparison of all possible nested models

The model with the final parameters combining LSM by VCTE, CAP and AST was compared to all possible nested model using the likelihood ratio test. AIC and BIC are provided for each model.

The likelihood ratio test was used for comparing the goodness of fit of two nested models. The null hypothesis (H0) of the test was that the more parsimonious model provided as good a fit as the model with the most parameters.

The table shows that the full model combining LSM by VCTE, CAP and AST is significantly better from each nested sub-model. In addition, it has the lowest AIC and BIC values.

	**Model LSM by VCTE + CAP + AST**	**Model LSM by VCTE + CAP**	**model LSM by VCTE + AST**	**model CAP+AST**	**model LSM by VCTE**	**model CAP**	**model AST**
**Likelihood ratio test**	-	$p=1 \times 10^{-8}$	$p=7 \times 10^{-5}$	$p=1 \times 10^{-5}$	$p=2 \times 10^{-10}$	$p=8 \times 10^{-14}$	$p=7 \times 10^{-11}$
**AIC**	408·4	438·9	422·3	425·7	449·0	464·8	451·2
**BIC**	423·8	450·5	433·9	437·3	456·8	472·5	458·9

AIC: Akaike information criterion, AST: aspartate aminotransferase, BIC: Bayesian information criterion, CAP: controlled attenuation parameter, LSM: liver stiffness measurement, VCTE: vibration-controlled transient elastography

Supplementary Table 6 – FAST cut-offs in the derivation cohort for a sensitivity ≥ 0·90 and a specificity ≥ 0·90 and associated diagnostic performance, for the identification of patients with NASH+NAS≥4+F≥2

When using the dual-cut-off approach, a set of two cut-offs were determined to rule in or rule out patients. The lower cut-off (rule-out cut-off) corresponded to a cut-off for sensitivity ≥ 0.90. The higher cut-off (rule-in cut-off) corresponded to a cut-off for specificity ≥ 0.90.

The table provides the two cut-offs for a sensitivity ≥ 0.90 and a specificity ≥ 0.90 and their associated diagnostic performance in terms of sensitivity, specificity, positive and negative predictive values and positive and negative likelihood ratios.

	**Cut-off for Se≥0·90**	**Cut-off for Sp≥0·90**
**FAST score cut-off**	0·35	0·67
**Se (95%CI)**<br>**TP/(TP+FN)**<br><br>**Sp (95%CI)**<br>**TN/(TN+FP)**	Se = 0·90 (0·85-0·94)<br>157/(157+17)<br><br>Sp = 0·53 (0·45-0·60)<br>93/(93+83)	Se = 0·48 (0·41-0·56)<br>84/(84+90)<br><br>Sp = 0·90 (0·85-0·94)<br>159/(159+17)
**PPV (95% CI)**<br>**TP/(TP+FP)**<br><br>**NPV (95% CI)**<br>**TN/(TN+FN)**	PPV = 0·65 (0·58-0·77)<br>157/(157+83)<br><br>NPV = 0·85 (0·77-0·88)<br>93/(93+17)	PPV = 0·83 (0·75-0·87)<br>84/(84+17)<br><br>NPV = 0·64 (0·56-0·76)<br>159/(159+90)
**LR+ (95% CI)**<br><br>**LR- (95% CI)**	LR+ = 1·91 (1·62-2·25)<br><br>LR- = 0·18 (0·12-0·30)	LR+ = 5·00 (3·10-8·06)<br><br>LR- = 0·57 (0·49-0·67)

CI: confidence interval, FN: number of false negative, FP: number of false positive, LR-: negative likelihood ratio, LR+: positive likelihood ratio, NPV: negative predictive value, PPV: positive predictive value, Se: sensitivity, Sp: specificity, TN: number of true negative, TP: number of true positive.

Supplementary Table 7 – Performance comparison of the FAST score with FIB-4 and NFS using the dual-cut-off approach (0·35 and 0·67 for FAST, 1·30 and 3·25 for FIB-4 and -1·455 and 0·676 for NFS), in the derivation and external validation cohorts

FAST performance for the diagnostic of NASH+NAS≥4+F≥2 were compared to FIB-4 and NFS in terms of AUROC and when using the dual-cut-off approach. Cut-offs used were cut-offs for the derivation cohort for FAST and published cut-offs for FIB-4 and NFS.[3,4]

For each lower (rule-out) cut-off, corresponding specificity, negative predictive value and negative likelihood ratio were computed. For each higher (rule-in) cut-off, corresponding sensitivity, positive predictive value and positive likelihood ratio were computed. The proportion in-between the rule-in and rule-out cut-offs (grey zone) was also computed. AUROC comparison was performed using the Delong test.

Of note the cases where both sensitivity and positive predictive value were equal to zero corresponded to cases where only false positive patients were found above the specified threshold. The cases where the sensitivity was equal to zero and the positive predictive value was equal to NaN (not a number) corresponded to the case where all score values were below the specified threshold.

		Derivation cohort	French bariatric surgery cohort	USA screening cohort	China Hong-Kong NAFLD cohort	China Wenzhou NAFLD cohort	French NAFLD cohort	Malaysian NAFLD cohort	Turkish NAFLD cohort	Pooled external patients cohort
	**n**	339	75	233	83	104	182	175	129	981
	**Prevalence of NASH+NAS≥4+F≥2**	168/339 (50%)	12/75 (16%)	28/233 (12%)	36/83 (43%)	9/104 (9%)	78/182 (43%)	35/175 (20%)	74/129 (57%)	272/981 (28%)
**FAST**	**AUROC (95% CI)**	0·80 (0·75-0·84)	0·94 (0·89-0·99)	0·86 (0·80-0·92)	0·85 (0·76-0·93)	0·84 (0·73-0·95)	0·80 (0·73-0·86)	0·85 (0·78-0·91)	0·74 (0·65-0·82)	0·85 (0·82-0·87)
	**Se (at FAST score=0·67) Sp (at FAST score=0·35)**	Se=0·49 (82/168) Sp=0·52 (89/171)	Se=0·67 (8/12) Sp=0·76 (48/63)	Se=0·25 (7/28) Sp=0·85 (174/205)	Se=0·58 (21/36) Sp=0·55 (26/47)	Se=0·44 (4/9) Sp=0·56 (53/95)	Se=0·45 (35/78) Sp=0·56 (58/104)	Se=0·60 (21/35) Sp=0·54 (75/140)	Se=0·49 (36/74) Sp=0·35 (19/55)	Se=0·49 (132/272) Sp=0·64 (453/709)
	**PPV (at FAST score=0·67) NPV (at FAST score=0·35)**	PPV=0·83 (82/99) NPV=0·84 (89/106)	PPV=0·67 (8/12) NPV=1·00 (48/48)	PPV=0·78 (7/9) NPV=0·95 (174/184)	PPV=0·81 (21/26) NPV=0·93 (26/28)	PPV=0·33 (4/12) NPV=0·98 (53/54)	PPV=0·76 (35/46) NPV=0·87 (58/67)	PPV=0·54 (21/39) NPV=0·97 (75/77)	PPV=0·78 (36/46) NPV=0·73 (19/26)	PPV=0·69 (132/190) NPV=0·94 (453/484)
	**LR+ (at FAST score=0·67) LR- (at FAST score=0·35)**	LR+=4·91 LR-=0·19	LR+=10·50 LR-=0·00	LR+=25·62 LR-=0·42	LR+=5·48 LR-=0·10	LR+=5·28 LR-=0·20	LR+=4·24 LR-=0·21	LR+=4·67 LR-=0·11	LR+=2·68 LR-=0·27	LR+=5·93 LR-=0·18

	**Grey zone (FAST between 0·35 and 0·67)**	131/339 (39%)	15/75 (20%)	39/233 (17%)	29/83 (35%)	37/104 (36%)	69/182 (38%)	58/175 (33%)	57/129 (44%)	304/981 (31%)
**FIB-4**	**AUROC**	0·70 (0·65-0·76)	0·82 (0·70-0·94)	0·70 (0·60-0·80)	0·76 (0·65-0·87)	0·58 (0·33-0·83)	0·63 (0·55-0·71)	0·80 (0·71-0·89)	0·75 (0·67-0·84)	0·74 (0·70-0·77)
	**AUROC comparison vs FAST**	p=0·0019	p=0·054	p=0·00011	p=0·16	p=0·013	p=0·00016	p=0·29	p=0·79	p=2 x 10^{-9}
	**Se (at FIB-4=3·25) Sp (at FIB-4=1·30)**	Se=0·08 (14/168) Sp=0·75 (129/171)	Se=0·00 (0/12) Sp=0·92 (58/63)	Se=0·00 (0/28) Sp=0·76 (155/205)	Se=0·11 (4/36) Sp=0·70 (33/47)	Se=0·00 (0/9) Sp=0·81 (77/95)	Se=0·09 (7/78) Sp=0·53 (55/104)	Se=0·03 (1/35) Sp=0·83 (116/140)	Se=0·08 (6/74) Sp=0·76 (42/55)	Se=0·07 (18/272) Sp=0·76 (536/709)
	**PPV (at FIB-4=3·25) NPV (at FIB-4=1·30)**	PPV=0·88 (14/16) NPV=0·65 (129/197)	PPV=NaN NPV=0·89 (58/65)	PPV=NaN NPV=0·91 (155/170)	PPV=0·80 (4/5) NPV=0·77 (33/43)	PPV=0·00 (0/2) NPV=0·93 (77/83)	PPV=0·88 (7/8) NPV=0·65 (55/84)	PPV=0·25 (1/4) NPV=0·92 (116/126)	PPV=1·00 (6/6) NPV=0·57 (42/74)	PPV=0·72 (18/25) NPV=0·83 (536/645)
	**LR+ (at FIB-4=3·25) LR- (at FIB-4=1·30)**	LR+=7·12 LR-=0·54	LR+=NaN LR-=0·63	LR+=NaN LR-=0·71	LR+=5·22 LR-=0·40	LR+=0·00 LR-=0·82	LR+=9·33 LR-=0·70	LR+=1·33 LR-=0·34	LR+=Inf LR-=0·57	LR+=6·70 LR-=0·53
	**Grey zone (FIB-4 between 1·30 and 3·25)**	126/339 (37%)	10/75 (13%)	63/233 (27%)	35/83 (42%)	19/104 (18%)	90/182 (49%)	45/175 (26%)	49/129 (38%)	311/981 (32%)
**NFS**	**AUROC (95% CI)**	0·69 (0·63-0·75)	0·62 (0·41-0·82)	0·67 (0·57-0·77)	0·62 (0·50-0·74)	0·53 (0·25-0·81)	0·66 (0·58-0·74)	0·77 (0·68-0·86)	0·67 (0·57-0·76)	0·68 (0·65-0·72)
	**AUROC comparison vs FAST**	p=0·0021	p=0·0016	p=0·00088	p=0·0022	p=0·016	p=0·0031	p=0·16	p=0·26	p=2 x 10^{-13}
	**Se (at NFS=0·676) Sp (at NFS=-1·455)**	Se=0·17 (29/168) Sp=0·53 (90/171)	Se=0·33 (4/12) Sp=0·35 (22/63)	Se=0·18 (5/28) Sp=0·38 (77/205)	Se=0·17 (6/36) Sp=0·43 (20/47)	Se=0·00 (0/9) Sp=0·85 (81/95)	Se=0·31 (24/78) Sp=0·33 (34/104)	Se=0·06 (2/35) Sp=0·76 (107/140)	Se=0·14 (10/74) Sp=0·53 (29/55)	Se=0·19 (51/272) Sp=0·52 (370/709)
	**PPV (at NFS=0·676) NPV (at NFS==-1·455)**	PPV=0·69 (29/42) NPV=0·69 (90/131)	PPV=0·36 (4/11) NPV=0·88 (22/25)	PPV=0·19 (5/27) NPV=0·93 (77/83)	PPV=0·86 (6/7) NPV=0·69 (20/29)	PPV=0·00 (0/1) NPV=0·93 (81/87)	PPV=0·62 (89/143) NPV=0·81 (34/42)	PPV=0·50 (2/4) NPV=0·89 (107/120)	PPV=0·83 (10/12) NPV=0·58 (29/50)	PPV=0·50 (51/101) NPV=0·85 (370/436)
	**LR+ (at NFS=0·676) LR- (at NFS==-1·455)**	LR+=2·27 LR-=0·46	LR+=3·00 LR-=0·72	LR+=1·66 LR-=0·57	LR+=7·83 LR-=0·59	LR+=0·00 LR-=0·78	LR+=2·13 LR-=0·31	LR+=4·00 LR-=0·49	LR+=3·72 LR-=0·54	LR+=2·66 LR-=0·46

	**Grey zone (NFS between -1·455 and 0·676)**	166/339 (49%)	39/75 (52%)	123/233 (53%)	47/83 (57%)	16/104 (15%)	101/182 (55%)	51/175 (29%)	67/129 (52%)	444/981 (45%)

AUROC: area under the receiver operating curve, CI: confidence interval, F: fibrosis stage, FIB-4: fibrosis-4 index, LR-: negative likelihood ratio, LR+: positive likelihood ratio, NAFLD: non-alcoholic fatty liver disease, NaN: not a number, NAS: NAFLD activity score, NASH: non-alcoholic steatohepatitis, NFS: NAFLD fibrosis score, NPV: negative predictive value, PPV: positive predictive value, Se: sensitivity, Sp: specificity, USA: United States of America.

Supplementary Table 8 – Comparison of diagnostic performance of the FAST score and the score combining LSM by VCTE, CAP, AST and DM or HTN

Extra models combining LSM by VCTE, CAP and AST with DM or HTN were constructed. No parameter selection was performed since the parameters for the FAST score were combined with either DM or HTN. Each score was developed the same way as the FAST score, in the same derivation cohort.

The AUROC of each score (the FAST score, the score combining LSM by VCTE, CAP, AST and DM and the score combining LSM by VCTE, CAP, AST and HTN) were appraised in each cohort (the derivation cohort and all external validation cohort). Area under the receiver operating curve comparison was performed using the Delong test.

	Derivation cohort	French bariatric surgery cohort	USA screening cohort	China Hong-Kong NAFLD cohort	China Wenzhou NAFLD cohort	French NAFLD cohort	Malaysian NAFLD cohort	Turkish NAFLD cohort	Pooled external patients cohort
N	339	75	233	83	104	182	175	129	981
Prevalence of NASH+NAS≥4+F≥2	168/339 (50%)	12/75 (16%)	28/233 (12%)	36/83 (43%)	9/104 (9%)	78/182 (43%)	35/175 (20%)	74/129 (57%)	272/981 (28%)
**AUROC (95% CI) for FAST score (LSM by VCTE + CAP + AST)**	0·80 (0·76-0·85)	0·95 (0·91-0·99)	0·86 (0·80-0·93)	0·85 (0·76-0·93)	0·84 (0·73-0·95)	0·80 (0·73-0·86)	0·85 (0·78-0·91)	0·74 (0·65-0·82)	0·85 (0·83-0·87)
**AUROC (95% CI) for the score LSM by VCTE + CAP + AST + DM**	0·83 (0·79-0·87)	0·93 (0·89-0·98)	0·86 (0·79-0·93)	0·83 (0·74-0·92)	0·83 (0·70-0·95)	0·79 (0·73-0·86)	0·86 (0·80-0·92)	0·73 (0·65-0·82)	0·85 (0·82-0·87)
**AUROC comparison vs FAST**	p=0·051	p=0·30	p=0·62	p=0·39	p=0·68	p=0·76	p=0·32	p=0·86	p=0·92
**AUROC (95% CI) for the score LSM by VCTE + CAP + AST + HTN**	0·81 (0·77-0·86)	0·95 (0·91-0·99)	0·85 (0·78-0·92)	0·82 (0·72-0·91)	0·83 (0·71-0·94)	–	0·85 (0·79-0·92)	0·75 (0·67-0·84)	0·86 (0·84-0·89)
**AUROC comparison vs FAST**	p=0·21	p=0·70	p=0·20	p=0·0062	p=0·18	–	p=0·36	p=0·18	p=0·43

AUROC: area under the receiver operating curve, CAP: controlled attenuation parameter, CI: confidence interval, DM: diabetes mellitus, HTN: hypertension, LSM: liver stiffness measurement, NAFLD: non-alcoholic fatty liver disease, VCTE: vibration-controlled transient elastography, USA: United States of America.

Supplementary Table 9 – FAST cut-offs for a sensitivity ≥ 0·90 and a specificity ≥ 0·90 and associated diagnostic performance, for the identification of patients with NASH+NAS≥4+F≥3

	**Cut-off for Se≥0·90**	**Cut-off for Sp≥0·90**
**FAST score Cut-off**	0·38	0·76
**Se (95%CI)**<br>**TP/(TP+FN)**<br>**Sp (95%CI)**<br>**TN/(TN+FP)**	Se = 0·91 (0·84-0·95)<br>98/(98+10)<br>Sp = 0·47 (0·41-0·54)<br>114/(114+128)	Se = 0·42 (0·32-0·52)<br>45/(45+63)<br>Sp = 0·92 (0·88-0·95)<br>223/(223+19)
**PPV (95% CI)**<br>**TP/(TP+FP)**<br>**NPV (95% CI)**<br>**TN/(TN+FN)**	PPV = 0·43 (0·37-0·62)<br>98/(98+128)<br>NPV = 0·92 (0·86-0·94)<br>114/(114+10)	PPV = 0·70 (0·60-0·78)<br>45/(45+19)<br>NPV = 0·78 (0·70-0·86)<br>(223/(223+63)
**LR+ (95% CI)**<br>**LR- (95% CI)**	LR+ = 1·72 (1·50-1·96)<br>LR- = 0·20 (0·11-0·36)	LR+ = 5·31 (3·26-8·63)<br>LR- = 0·63 (0·54-0·75)

CI: confidence interval, FN: number of false negative, FP: number of false positive, LR-: negative likelihood ratio, LR+: positive likelihood ratio, NPV: negative predictive value, PPV: positive predictive value, Se: sensitivity, Sp: specificity, TN: number of true negative, TP: number of true positive.

Supplementary Table 10: Diagnostic performance of the FAST score for the diagnostic of NASH+NAS≥4+F≥3 in the derivation and external validation cohorts

	AUROC (95% CI)	n	Prevalence of NASH+NAS≥4+F≥3	Performance using dual cut-off (cut-offs from derivation cohort)		
				rule-out zone	grey zone	rule-in zone
**Derivation cohort**	0·79 (0·74-0·84)	350	108/350 (31%)	**FAST≤0·38** n=128/350 (37%) Se=0·91 (98/108) Sp=0·47 (114/242) NPV=0·92 (114/124)	**FAST: 0·38-0·76** n=158/350 (45%)	**FAST>0·76** n=64/350 (18%) Sp=0·92 (223/242) Se=0·42 (45/108) PPV=0·70 (45/64)
**French bariatric surgery cohort**	0·94 (0·89-0·99)	110	10/110 (9%)	**FAST<0·38** n=69/110 (63%) Se=1·00 (10/10) Sp=0·69 (69/100) NPV=1·00 (69/69)	**FAST: 0·38-0·76** n=31/110 (28%)	**FAST≥0·76** n=10/110 (9%) Sp=0·96 (96/100) Se=0·60 (6/10) PPV=0·60 (6/10)
**USA screening cohort**	0·88 (0·79-0·98)	242	10/242 (4%)	**FAST<0·38** n=200/242 (83%) Se=0·60 (6/10) Sp=0·83 (193/232) NPV=0·98 (193/197)	**FAST: 0·38-0·76** n=39/242 (16%)	**FAST≥0·76** n=3/242 (1%) Sp=1·00 (231/232) Se=0·20 (2/10) PPV=0·67 (2/3)
**China Hong-Kong NAFLD cohort**	0·82 (0·71-0·92)	83	26/83 (31%)	**FAST<0·38** n=30/83 (36%) Se=0·92/24/26) Sp=0·47 (27/57) NPV=0·93 (27/29)	**FAST: 0·38-0·76** n=42/83 (51%)	**FAST≥0·76** n=11/83 (13%) Sp=0·95 (54/57) Se=0·31 (8/26) PPV=0·73 (8/11)
**China Wenzhou NAFLD cohort**	0·88 (0·78-0·99)	104	3/104 (3%)	**FAST<0·38** n=59/104 (57%) Se=1·00 (3/3) Sp=0·57 (58/101) NPV=1·00 (58/58)	**FAST: 0·38-0·76** n=42/104 (40%)	**FAST≥0·76** n=3/104 (3%) Sp=0·97 (98/101) Se=0·00 (0/3) PPV=0·00 (0/3)
**French NAFLD cohort**	0·83 (0·77-0·89)	182	49/182 (27%)	**FAST<0·38** n=75/182 (41%) Se=0·92 (45/49) Sp=0·51 (68/133) NPV=0·94 (68/72)	**FAST: 0·38-0·76** n=80/182 (44%)	**FAST≥0·76** n=27/182 (15%) Sp=0·94 (125/133) Se=0·39 (19/49) PPV=0·70 (19/27)

**Malaysian NAFLD cohort**	0·83 (0·75-0·92)	176	26/176 (15%)	**FAST<0·38**<br>n=81/176 (46%)<br>Se=0·88 (23/26)<br>Sp=0·52 (78/50)<br>NPV=0·96 (78/81)	**FAST: 0·38-0·76**<br>n=72/176 (41%)	**FAST≥0·76**<br>n=23/176 (13%)<br>Sp=0·93 (139/150)<br>Se=0·46 (12/26)<br>PPV=0·52 (12/23)
**Turkish NAFLD cohort**	0·73 (0·64-0·83)	129	45/129 (35%)	**FAST<0·38**<br>n=31/129 (24%)<br>Se=0·89 (40/45)<br>Sp=0·29 (24/84)<br>NPV=0·83 (24/29)	**FAST: 0·38-0·76**<br>n=70/129 (54%)	**FAST≥0·76**<br>n=28/129 (22%)<br>Sp=0·89 (75/84)<br>Se=0·42 (19/45)<br>PPV=0·68 (19/28)
**Pooled external patients cohort**	0·86 (0·83-0·89)	1026	169/1026 (16%)	**FAST<0·38**<br>n=545/1026 (53%)<br>Se=0·89 (151/169)<br>Sp=0·60 (517/857)<br>NPV=0·97 (517/535)	**FAST: 0·38-0·76**<br>n=376/1026 (37%)	**FAST≥0·76**<br>n=105/1026 (10%)<br>Sp=0·95 (818/857)<br>Se=0·39 (66/169)<br>PPV=0·63 (66/105)

AUROC: area under the receiver operating curve, NALFD: non-alcoholic fatty liver disease, NASH: non-alcoholic fatty liver disease: NAS: NAFLD activity score, NPV: negative predictive value, PPV: positive predictive value, Se: sensitivity, Sp: specificity, USA: United States of America.

Supplementary Table 11: Comparison of FAST score, FAST score individual parameters and histological parameters between correctly classified and misclassified patients in the rule-out and rule-in zones

	Rule-out zone (FAST≤0.35)			Rule-in zone (FAST≥0.67)		
	**False negative**	**True negative**	**P value**	**False positive**	**True positive**	**P value**
**n**	18	95	–	17	84	–
**FAST score**	0·29 (0·16-0·30)	0·19 (0·12-0·30)	0·18	0·78 (0·73-0·85)	0·80 (0·72-0·89)	0·28
**LSM by VCTE**	6·2 (4·6-11·3)	6·1 (4·8-8·0)	0·45	18·8 (12·5-23·1)	17·6 (11·8-26·3)	0·72
**CAP**	334 (294-340	312 (275-342)	0·45	369 (318-400)	370 (348-394)	0·81
**AST**	24·0 (22·0-28·8)	24·0 (20·0-30·5)	0·92	43·0 (36·0-59·0)	55·5 (44·0-70·0)	0·12
**Fibrosis stage**			2×10^{-12}			$8 \times 10^{-}$
**0**	0/18 (0%)	39/95 (41%)		2/17 (12%)	0/84 (0%)	
**1**	0/18 (0%)	43/95 (45%)		7/17 (41%)	0/84 (0%)	
**2**	10 /18 (56%)	6/95 (6%)		2/17 (12%)	23/84 (27%)	
**3**	7/18 (39%)	6/95 (6%)		4/17 (24%)	42/84 (50%)	
**4**	1/18 (6%)	1/95 (1%)		2/17 (12%)	19/84 (23%)	
**Ballooning grade**			2×10^{-7}			1×10^{-9}
**0**	0/18 (0%)	48/95 (51%)		7/17 (41%)	0/84 (0%)	
**1**	8/18 (44%)	40/95 (42%)		9/17 (53%)	25/84 (30%)	
**2**	10/18 (56%)	7/95 (7%)		1/17 (6%)	59/84 (70%)	
**Lobular inflammation**			1×10^{-7}			3.3×10^{-4}
**0**	0/18 (0%)	50/95 (53%)		3/17 (18%)	0/84 (0%)	
**1**	14/18 (78%)	45/95 (47%)		14/17 (82%)	57/84 (68%)	
**2**	3/18 (17%)	0/95 (0%)		0/17 (0%)	25/84 (30%)	
**3**	1/18 (6%)	0/95 (0%)		0/17 (0%)	2/84 (2%)	
**Steatosis grade**			0·022			5.0×10^{-4}
**0**	0/18 (0%)	16/95 (17%)				
**1**	4/18 (22%)	40/95 (42%)		8/17 (47%)	6/84 (7%)	
**2**	10 (56%)	26/95 (27%)		3/17 (18%)	29/84 (35%)	
**3**	4 (22%)	13/95 (14%)		6/17 (35%)	49/84 (58%)	
**NAS≥4**			5×10^{-9}			1×10^{-9}
**No**	0/18 (0%)	72/95 (76%)		10/17 (59%)	0/84 (0%)	
**Yes**	18/18 (100%)	23/95 (24%)		7 (41%)	84/84 (100%)	
**NASH**			2×10^{-7}			1×10^{-7}
**No**	0/18 (0%)	67/95 (69%)		8/17 (47%)	0/84 (0%)	
**Yes**	18/18 (100%)	28/95 (31%)		9/17 (53%)	84/84 (100%)	

Distribution is expressed as median (interquartile range: quartile 1- quartile 3) or figure (percentage). AST: aspartate aminotransferase, CAP: controlled attenuation parameter, FAST score: Fibroscan AST score, LSM: liver stiffness measurement, NAFLD: non-alcoholic fatty liver disease, NASH: non-alcoholic steatohepatitis, NAS: NAFLD activity score, VCTE: vibration-controlled transient elastography.

Supplementary Figure 1. Boxplot of FAST score in the derivation cohort versus (A) fibrosis stage, (B) steatosis grade, (C) lobular inflammation grade, (D) ballooning grade.

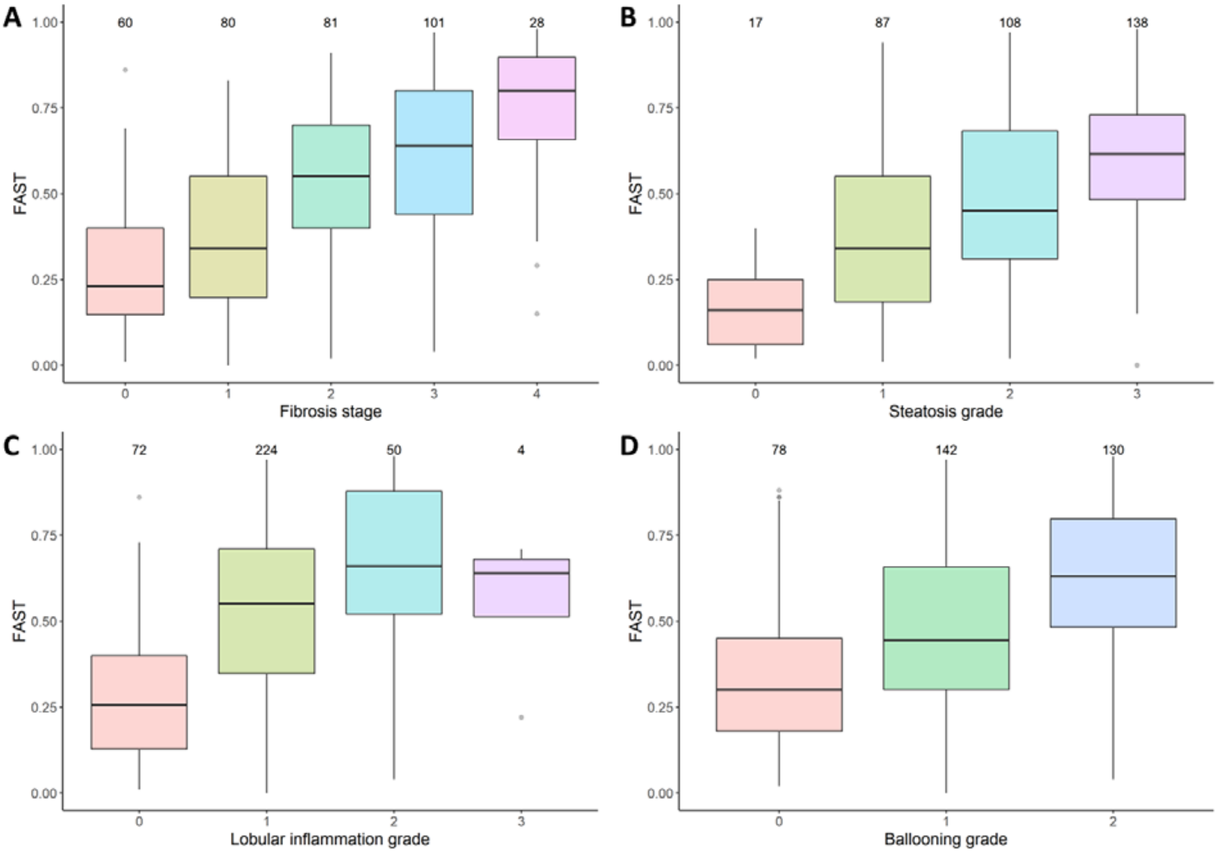

References

1. Kleiner DE, Brunt EM, Van Natta M, et al. Design and validation of a histological scoring system for nonalcoholic fatty liver disease. *Hepatology* 2005; **41**(6): 1313-21.
2. Bedossa P, Consortium FP. Utility and appropriateness of the fatty liver inhibition of progression (FLIP) algorithm and steatosis, activity, and fibrosis (SAF) score in the evaluation of biopsies of nonalcoholic fatty liver disease. *Hepatology* 2014; **60**(2): 565-75.
3. Sterling RK, Lissen E, Clumeck N, et al. Development of a simple noninvasive index to predict significant fibrosis in patients with HIV/HCV coinfection. *Hepatology* 2006; **43**(6): 1317-25.
4. Angulo P, Hui JM, Marchesini G, et al. The NAFLD fibrosis score: a noninvasive system that identifies liver fibrosis in patients with NAFLD. *Hepatology* 2007; **45**(4): 846-54.
